# Numerical modelling of static and dynamic factors controlling the progressive retreat of rocky coastal cliffs

**DOI:** 10.1007/s10346-026-02800-2

**Published:** 2026-06-27

**Authors:** F. Feliziani, G. M. Marmoni, M. Montagnese, R. Sobhani, D. Istrati, E. Eberhardt, S. Martino

**Affiliations:** 1 Sapienza University of Rome and CERI Research Centre for Geological Risks, Rome, Italy; 2 National Technical University of Athens, Athens, Greece; 3 Technical University of Braunschweig, Brunswick, Germany; 4 Aarhus University, Aarhus, Denmark; 5 The University of British Columbia, Vancouver, Canada

**Keywords:** Numerical modelling, FDEM, CFD, Progressive rock failure, Coastal landslides, Sea waves

## Abstract

This study investigates instability mechanisms in soft rocky coastal cliffs through an integrated numerical framework that couples computational fluid dynamics (CFD) with the finite-discrete element method (FDEM), applied to the tuffaceous cliffs of Ventotene Island (Italy). The modelling strategy systematically explores the role of structural predisposition, marine erosion, wave-induced loading, thermal forcing, and time-dependent strength degradation in controlling cliff stability and failure evolution. The results show that neither gravity loading nor basal undercutting alone can reproduce realistic failure mechanisms, even in the presence of a DFN. Progressive rock failure (PRF) emerges only when basal erosion is explicitly coupled with inward-migrating strength degradation, implemented using a simplified fatigue-based S–N approach. Under the considered lithology and the present Mediterranean climatic conditions, wave impacts and thermal forcing induce predominantly elastic or sub-critical mechanical responses and do not directly trigger failure. However, wave-induced dynamic perturbations concentrate displacements in fractured cliff sectors, suggesting a preparatory role through fatigue and stress redistribution, with potential triggering effects once critical degradation thresholds are approached. Thermal forcing becomes mechanically effective only under amplified temperature excursions or in lithologies with higher thermal sensitivity. The coupled CFD–FDEM approach is therefore interpreted as a process-based, exploratory framework that captures the dynamic response of coastal cliffs to realistic environmental boundary conditions, rather than as a direct predictor of wave-driven collapse. The methodology provides a physically grounded and transferable framework for investigating multi-forcing interactions, damage accumulation, and PRF in coastal cliffs, offering new insights into the mechanisms governing coastal instability and long-term cliff evolution.

**Supplementary Information:**

The online version contains supplementary material available at https://doi.org/10.1007/s10346-026-02800-2.

## Introduction

Rock coasts make up almost 80% of the world’s oceanic shorelines (Castedo et al. 2017), and approximately 50% of the world’s population lives within 60 km of the coast (Salman et al. 2004). In this framework, Kron (2013) reports that many of the most devastating natural disasters in terms of losses occurred near a coast, highlighting that coastal regions are the most threatened areas in the world by natural hazards. Among the aforementioned phenomena, coastal retreat processes and related landslides represent a significant source of geohazard (Alberti et al. 2022; Dixon and Bromhead 2002; Fiorucci et al. 2022; Leshchinsky et al. 2017; Marmoni and Martino 2025; Martino and Mazzanti 2014).

Coastal instability processes affecting rocky cliffs have been mostly investigated using predictive erosion models (Castedo et al. 2012; Hackney et al. 2013; Matsumoto et al. 2016; Sunamura 1982, 1977; Trenhaile 2011; Walkden and Hall 2005), analysing their geomorphological evolutionary pattern (Fairbridge 2004; Galone et al. 2024; Kennedy et al. 2014; Miccadei et al. 2019; Sunamura 2015; Torre et al. 2025), or focusing on landslide-induced tsunamis phenomena (Harbitz et al. 2006; Sassa 2023; Ward 2001). In this framework, numerical modelling-based studies dealing with the relationships between the predisposing, preparatory, and triggering factors (sensu Glade and Crozier 2005) that affect the mechanisms of sea cliff collapse are few.

The predisposing influence of cliff geometry and geomechanical conditions has been examined by Allison and Kimber (1998) and Shail et al. (1998), who highlighted how structural configuration governs stability. More recently, He et al. (2022) conducted a distinct element method (DEM) analysis of the Hell’s Mouth landslide with 3DEC, showing that the failure was primarily driven by the pre-existing fracture network and by the progressive undercutting of the cliff base.

The preparatory role of notch formation in sea cliff instability has also been the subject of numerical investigations. Calista et al. (2019) and Fazio et al. (2019) respectively used Plaxis and FLAC3D, demonstrating that basal notches play a key role in controlling the activation of translational, topple, and rockfall landslides. More recently, Napoli et al. (2025) explored the effect of cliff-notch slenderness on mechanical disturbance by performing a 2D finite element analysis using RS2. Despite these advances, most notch-related analyses rely on simplified material behaviour and do not fully capture progressive fracturing under cyclic or multi-physics forcing.

A significant step forward was the development of the theory of progressive rock failure -PRF- (Eberhardt et al. 1999, 1998; Martin and Chandler 1994; Scholz 1968), which provided a mechanistic framework for describing the initiation, accumulation, and migration of damage in brittle rock. This framework was subsequently applied to coastal cliffs by Styles et al. (2011), who employed the explicit finite-discrete element method (FDEM) code ELFEN to simulate the landslide of Joss Bay, reproducing the progressive upward development of strain originating from the basal notch. Lollino and Andriani (2017) later applied an FDEM approach using the Irazu code, demonstrating that fracture and fragmentation processes play a key role even under the low confinement conditions typical of coastal cliffs. More recently, Sutcliffe et al. (2025) employed the material point method (MPM) to model notch-driven cliff failure from fracture initiation through post-failure deformation.

Despite these advances, none of these studies explicitly incorporated the coupled effects of marine forcing, thermomechanical cycling, and weathering-driven strength degradation, processes that are known to exert a first-order control on progressive failure and cliff retreat in coastal environments.

Among these processes, wave action is recognised as one of the dominant marine controls on cliff dynamics; however, its role has been investigated mainly through erosive predictive models, with limited attention to how wave loading influences the deformational response of the rock mass. Among the most recent studies, Adams et al. (2005) identified the continuous wave breaking as a preparatory fatigue mechanism for the rock mass. However, Brain et al. (2014) suggested that, due to the very low magnitude of the strains and associated stresses generated by wave impact, microseismic ground motions are unlikely to drive sub-critical crack growth (Atkinson 1984) of microfractures unless the rock mass has already reached a critical pre-damaged state.

Other studies concerning the impact of sea waves on coastal systems have relied on physical modelling (e.g. Attili et al. 2023; Cuomo et al. 2010), hydrodynamic simulations (e.g. Altomare et al. 2015; Amini and Memari 2024; Hasanpour and Istrati 2022), or field measurements (e.g. Larroque et al. 2018). However, most of these were developed with a focus on coastal engineering structures. Applications to natural case studies are limited (e.g.Deng 2024; Khosravifardshirazi et al. 2025; Letortu et al. 2022; Shen et al. 2025; Sobhani et al. 2026), particularly those that explicitly simulate the deformational and damage response of a cliff. This highlights a persistent gap in linking hydrodynamic impact loads to the mechanical evolution and progressive damage of coastal rock slopes.

Thermal fatigue is widely recognised as a controlling factor for rock slope failures, both as a conditioning process that promotes progressive damage accumulation (Fiorucci et al. 2018; Gasc-Barbier et al. 2021; Grechi et al. 2021) and as a triggering mechanism (Gasc-Barbier et al. 2021). Although this process has been investigated through stress–strain numerical modelling (Alcaíno-Olivares et al. 2023; Gischig et al. 2011; Gunzburger et al. 2005; Marmoni et al. 2020; Morcioni et al. 2022), its role in coastal settings has received comparatively little attention. A similar limitation applies to the analysis of weathering processes. While weathering is widely recognised as an important driver of mechanical strength degradation in rocks (Eppes and Keanini 2017), its effects have been studied mainly through laboratory tests (e.g. Gupta and Rao 2000; Li et al. 2020; Nong and Towhata 2017; Tuǧrul 2004; Zhang et al. 2023), leaving a clear gap in their integration into numerical models simulating long-term cliff evolution under coupled multi-physics forcing. In this context, sub-critical crack growth represents a key preparatory mechanism, allowing fractures to propagate at stress intensities well below the rock’s critical threshold. Integrating these micro-scale processes into a unified stress–strain framework remains a significant challenge in long-term cliff evolution modelling.

Although some studies have modelled PRF in coastal cliffs, they primarily address this mechanism in relation to basal-notch undercutting. In contrast, the interaction between subaerial and marine-driven factors that controls the onset and evolution of progressive failure remains poorly understood (Rosser et al. 2013), and current stability models still lack an integrated treatment of these combined processes.

This study aims to provide new insights into the mechanisms driving coastal rock cliff collapse by numerically addressing the action of multiple environmental forcings. Rather than prescribing a fully coupled approach, the different controlling factors are investigated individually within a unified FDEM framework, with the objective of systematically isolating and quantifying the contribution of each process to the deformative response and damage evolution of the rock mass. Building on this strategy, the analyses also explore the cumulative effect of multiple forcings through simplified cyclic fatigue laws (i.e. a S–N curve), enabling the progressive degradation of mechanical properties to be reproduced within the same modelling framework. The research focuses on the sea cliff of Ventotene Island (Italy), due to its high exposure to wave impact and past evidence of instability. To this end, a multi-physics modelling approach is adopted, combining computational fluid dynamics (CFD) with FDEM stress–strain numerical modelling, in order to simulate the effects of wave loading, thermal cycling, and weathering/fatigue processes on cliff stability. The outcomes of this study are expected to enhance the understanding of preparatory mechanisms in rocky cliff collapse and contribute to the development of high-resolution predictive tools for coastal hazard assessment.

## Case study: the coastal cliffs of Ventotene Island

Ventotene is a small Mediterranean island located about 50 km off the Gulf of Gaeta on the western coast of Italy. Ventotene is primarily composed of pyroclastic deposits emplaced between 0.15 and 0.3 Ma, which dip gently north-eastward and unconformably overlie a sequence of basaltic to trachytic lavas (Gaeta et al. 2025; Metrich et al. 1988; Perrotta et al. 1996).

Coastal erosion represents a major geological hazard to Ventotene (Caiola et al. 1991). This process has occurred predominantly through cliff instabilities, mainly consisting of rockfalls, topples, and rock slides (sensu Hungr et al. 2014; Ruberti et al. 2020).

**Fig. 1:**
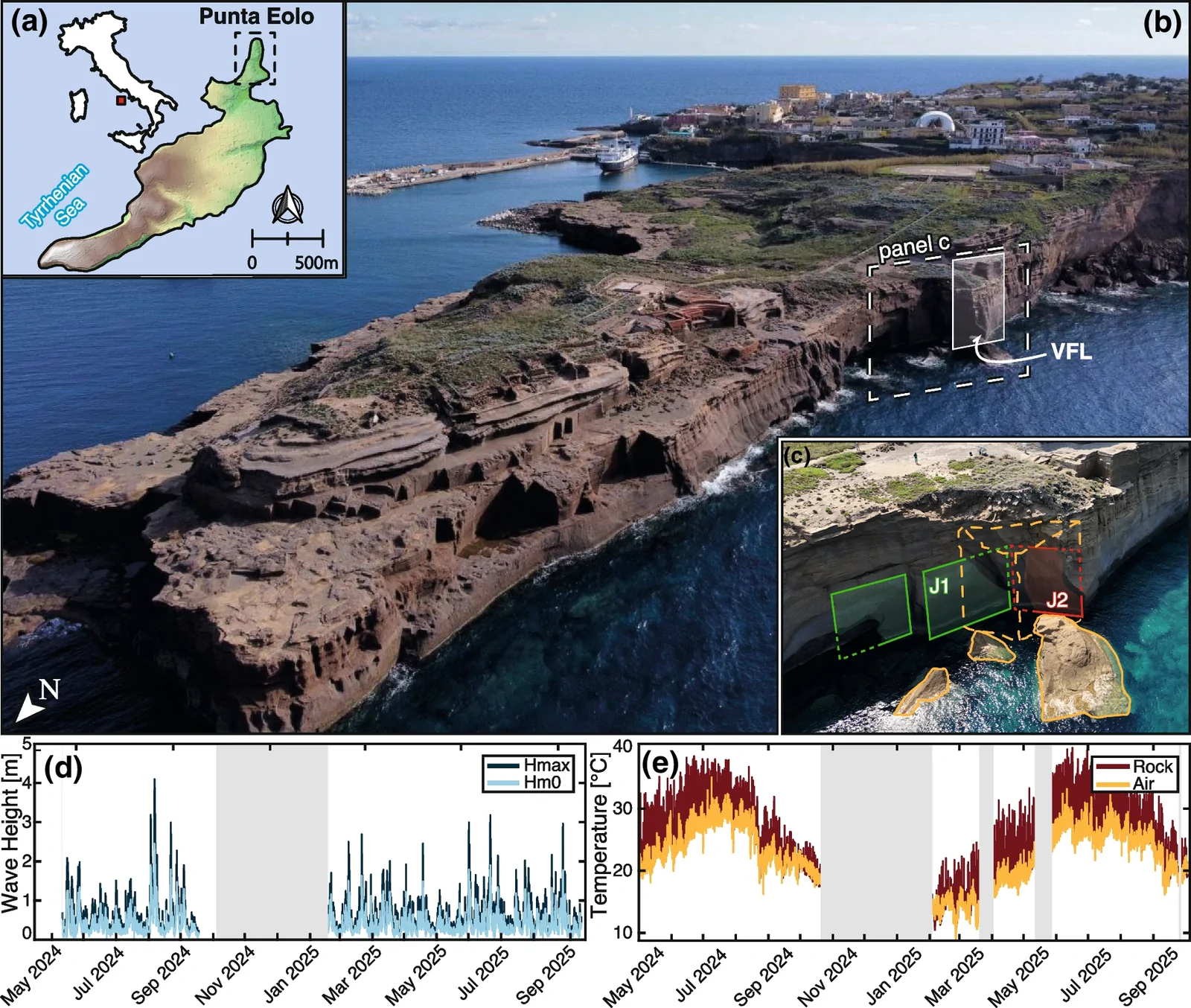
**a**) Geographic location of Ventotene Island. **b**) Aerial view of Punta Eolo promontory, with location of the Ventotene Field Lab (VFL). **c**) Detail of a toppled rock block detached at Punta Eolo in 2012. J1 and J2 joint sets are schematically represented, as well as the detached block (orange contour). **d**) Maximum (Hmax) and significant (Hm0) wave height and **e**) rock and air temperature time series recorded at Punta Eolo by the VFL

The study area is located at Punta Eolo Promontory, where frequent landslide events threaten the preservation of the Roman archaeological site of Villa Giulia (Ioannidis et al. 2024; Pegurri et al. 2025) (Fig. 1a, b). The geomechanical setting of the area is characterised by a primary joint set J0 (120/07°) related to bedding, and by secondary sets J1 (270/90 ± 5°), J2 (005/90 ± 5°), and J3 (090/90 ± 5°)—respectively located along the western, northern, and eastern coastal stretches of the promontory—associated with the progressive release of the rock mass caused by the aforementioned quarrying activities (Fig. 1c). These sub-vertical discontinuities are spaced 2 m apart and are often located near the cliff edge. Over the short term, small-scale rockfalls (< 0.1 m^3^) are the most common mechanism at Punta Eolo. They are concentrated around the convex faces and overhangs created by wind and wave impact on the cliff, and represent the primary driver of the ongoing coastal retreat in the area. Conversely, topple failures occur less frequently but typically involve larger rock volumes and contribute to more significant episodic retreat events.

The retreat process at Punta Eolo is particularly favoured by the high erodibility of the outcropping Punta Eolo Tuffs (PET) (Perrotta et al. 1996), which are classified as soft rock (i.e. UCS < 20 MPa; Brown 1981). In general, PET in the area are susceptible to weathering, similar to Neapolitan Yellow Tuff (Heap et al. 2018a; La Russa et al. 2017).

No data regarding the coastal retreat rate characterising the Punta Eolo cliff are available. However, analogous tuffaceous coastal slopes in neighbouring areas exhibit a coastal retreat rate between 0.05 and 1.28 m/year (Caputo et al. 2018; Di Luccio et al. 2023; Esposito et al. 2018, 2020; Massaro et al. 2023).

Since May 2024, the Ventotene Field Laboratory (VFL) has been operating at Punta Eolo to collect environmental and geotechnical data aimed at investigating coastal landslides by linking marine and subaerial controlling factors to the mechanical response of the rock mass (Feliziani et al. 2025). Instrumentation includes a fully equipped meteorological station to monitor weather conditions and a 1 MHz Acoustic Wave and Current Profiler (AWAC; Nortek Group) deployed on the seafloor 30 m offshore to measure wave dynamics. Wave statistics reveal peaks in the probability density at a significant wave height (Hm) of 0.2 m, which represent the conditions of the typical wave regime at Ventotene. However, open-sea wave regimes have also been recorded during strong winds coming from the West (255° N) that reach speeds of 85 km/h and cause significant wave heights of up to 4.6 m (Fig. 1d).

Thermal conditions at Ventotene are typical of a coastal Mediterranean climate, with minimum monthly mean temperatures of 9 °C in January and maxima of 26 °C in July–August (Caiola et al. 1991). The data recorded by the VFL reveal a minimum air temperature of 8.7°C in March and a maximum of 35.2 °C in July. Rock temperature time series consistently show higher values than air temperature due to the effect of direct solar radiation. Because the sensors are installed at a depth of approximately 9 cm, the recorded values represent damped thermal amplitudes, and even higher temperature fluctuations can be expected at the exposed cliff surface. The coastally mitigated seasonal thermal regime therefore induces rock-surface temperature oscillations ranging from 10.4 °C in February to 38.8 °C in July (Fig. 1e).

## Methods

**Fig. 2:**
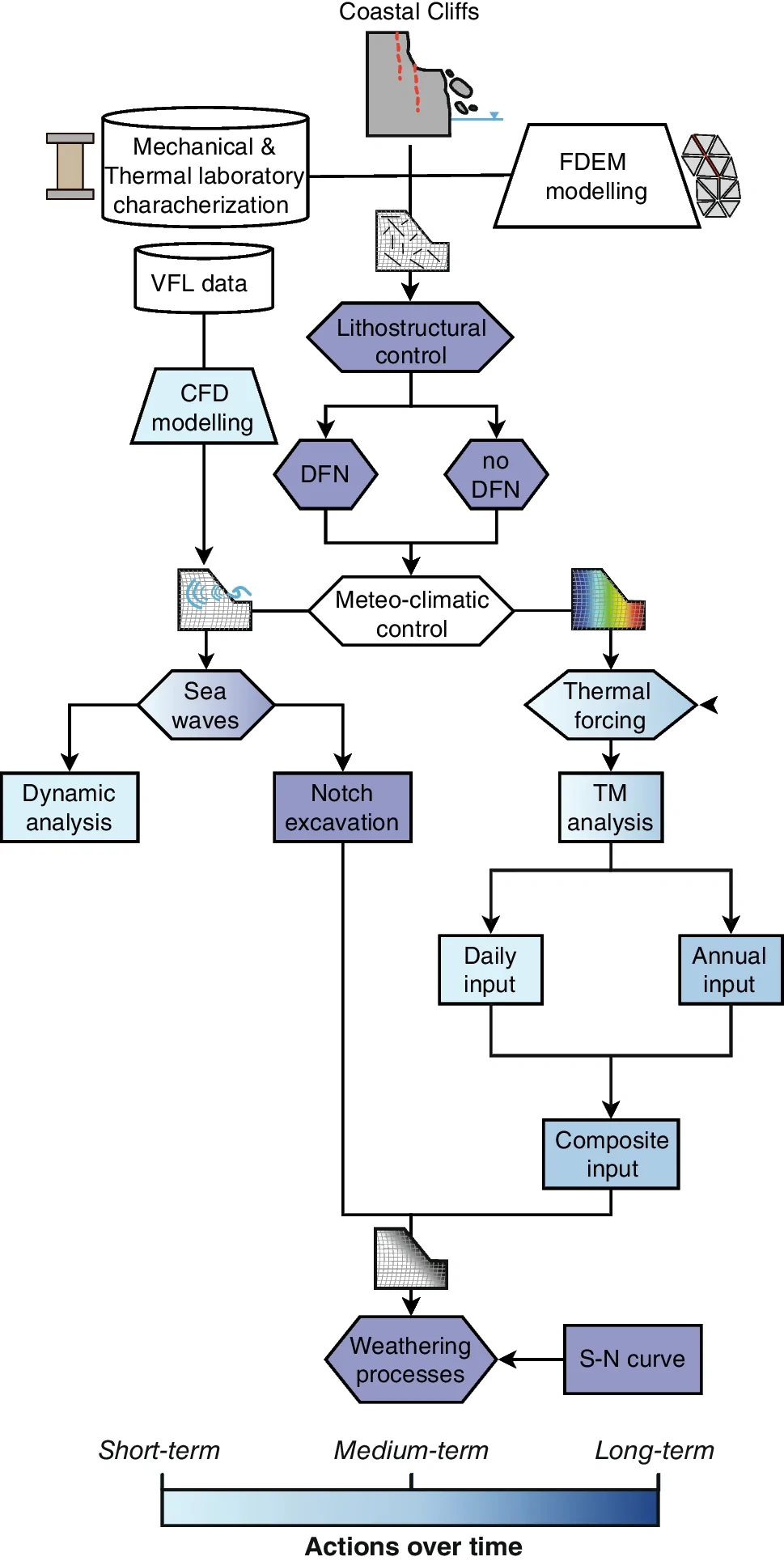
Methodological workflow adopted in the present research for coastal cliff stability analysis. The time at which the various control factors act is indicated by the colour scale used

The methodology aimed to evaluate the combined role of predisposing lithostructural features and meteo-climatic preparatory factors on cliff stability (Fig. 2). The approach integrated laboratory characterisation, numerical modelling, and field monitoring data from the VFL. The latter provides information on the forcing factors currently acting on the study site and on the site’s response. The FDEM solution was employed as the numerical tool, using mechanical, physical, and thermal properties obtained from laboratory tests on the tuffaceous units characterising the Ventotene case study. Preliminary static analyses evaluated the lithostructural control exerted by a discrete fracture network (DFN) on cliff stability. Two structural scenarios were considered: cliffs with DFN and cliffs without significant fracture systems (no DFN). This analysis stage was conducted to study how long-term (multi-year) conditions influence the evolution of the cliff system.

Two main environmental forcing mechanisms were considered: sea waves and thermal forcing. Thermal forcing was investigated through thermomechanical analyses using different thermal input signals referred to i) climatic conditions at the Ventotene case study, and ii) increased temperature amplitudes. The temperature-related climate action was therefore analysed in the context of medium-term (multi-day to seasonal) forcings.

Sea-wave impacts were analysed using data from both CFD modelling and data recorded by the VFL. The wave forcing was examined through a dynamic analysis to assess instantaneous loading effects, and the progressive notch excavation to simulate long-term erosive action at the cliff base.

Finally, to represent the cumulative degradation induced by both wave action and thermal cycling, the FDEM analyses incorporated a fatigue-based approach using S–N curves. This approach simulated the progressive decay of mechanical properties under repeated environmental loading, thereby capturing the synergistic effects of multiple cyclical climatic factors on long-term cliff stability.

## FDEM numerical model setup and input parameters

A FDEM modelling approach was adopted to analyse the brittle failure and PRF mechanism characterising the coastal cliff of Punta Eolo. Unlike other continuum- and discontinuum-based methods, the hybrid approach offered by the FDEM can explicitly simulate the transition from a continuum to a discontinuum with the insertion of new discontinuities (Lisjak and Grasselli 2014; Munjiza et al. 1995). In this framework, the 2D FDEM code Irazu with thermal coupling (Geomechanica Inc.) was employed. The modelling domain is discretised into triangular finite elements (TFEs) connected by cohesive crack elements (CCEs). Under linear elastic deformation, the model operated until excessive deformation caused CCEs to break under modes I and II and mixed-mode, corresponding with tensile, shear, and mixed fracture initiation processes, respectively.

Some additional details of the FDEM approach are provided in the Supplementary Material.

**Fig. 3:**
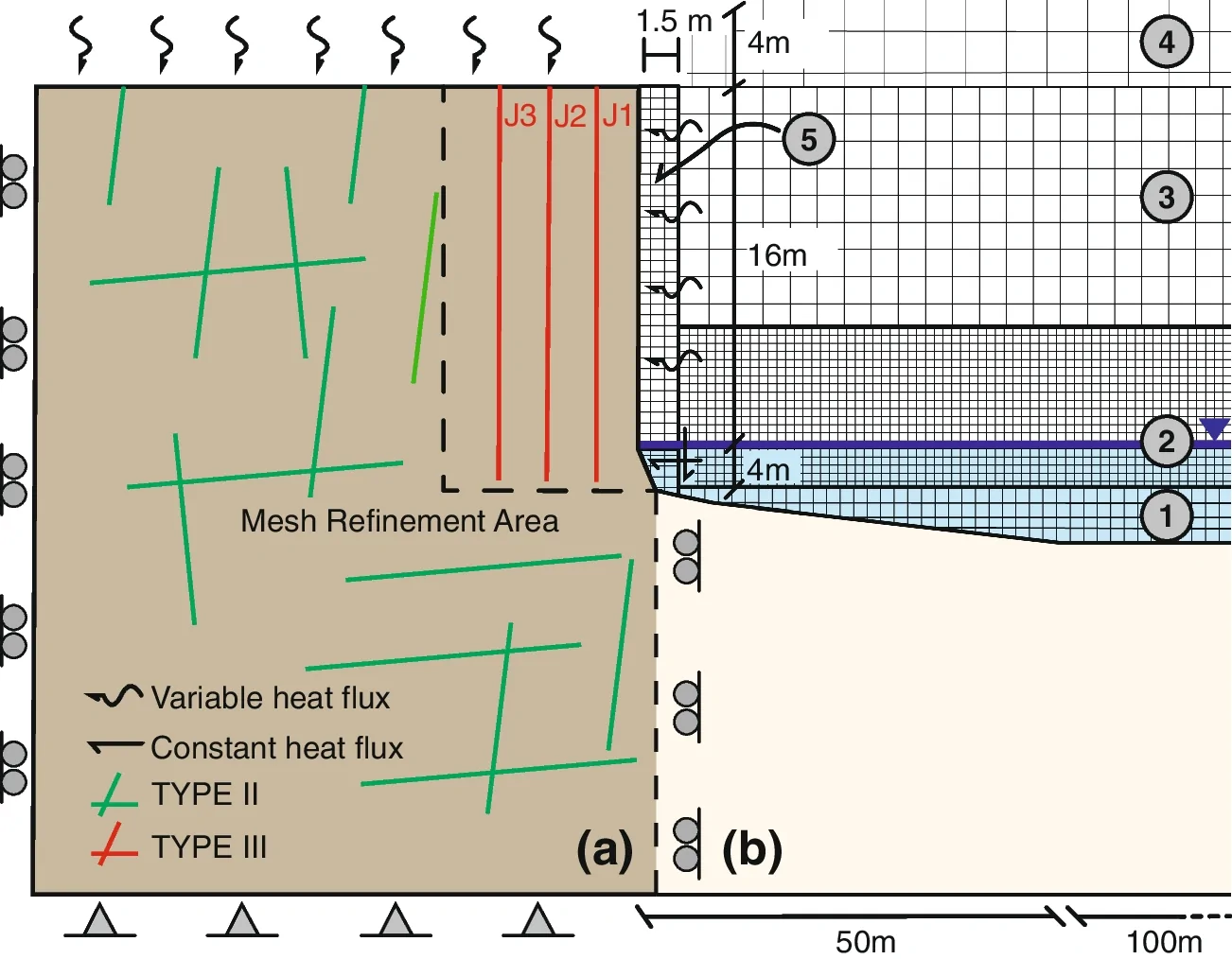
**a**) General 2D numerical domain setup designed for the FDEM modelling. Mechanical and thermal boundary conditions are reported, as well as the TYPE II and TYPE III geomechanical configurations. **b**) Computational domain used in the hydrodynamic simulation, derived from the site bathymetry and cliff topography. The circled numbers indicate the different domain regions characterised by different mesh sizes used

The landslide process was analysed using a simplified 2D topographic profile representative of the Punta Eolo cliff geometry (Fig. 3). The use of a representative section allows key controlling variables governing failure evolution to be isolated and examined in a controlled manner, while providing a valuable reference model for analogous coastal cliff settings. A 2D plane-strain formulation was assumed and considered appropriate because the analysed coastal cliff sector is laterally continuous and lacks significant three-dimensional irregularities.

Based on the average height of the western cliff of the investigated promontory, a 20-m-high profile was subsequently reproduced in Irazu. The cliff face is near-vertical, except for the lowermost 2 m that transitions to the horizontal seafloor at a 70° angle. This inclination further avoids meshing artefacts and reduces artificial stress concentrations at the cliff-seafloor intersection. A sea level depth of 4 m was assumed based on local bathymetry data (Feliziani et al. 2024), and the corresponding hydrostatic pressure was applied to the submerged slope face and seafloor. Groundwater within the rock mass was not modelled in order to isolate the mechanical response induced by external marine and atmospheric forcings only. The problem domain was discretised into approximately 0.6 M TFEs. A mesh refinement zone with a nominal finite element size of 0.05 m was defined along the cliff face and near-surface region (i.e. where fracturing is most likely to occur), ensuring accurate representation of fracture initiation and propagation trajectories (Munjiza and John 2002). The mesh was then gradually coarsened toward the model boundaries, where a nominal finite element size of 0.5 m was adopted to maintain computational efficiency.

The outcropping rock at Punta Eolo consists of a porous, matrix-supported tuff containing abundant coarse lithic clasts. Uniaxial compressive strength (UCS), Young’s modulus (*E*), and unit weight and porosity measurements were conducted in accordance with ISRM standards to define and derive the properties of the intact rock matrix (Table 1). In the absence of direct cohesion and tensile strength measurements, these were estimated as 25% and 10% of the UCS strength, respectively, following standard engineering practice and Irazu modelling guidelines (Geomechanica Inc. 2024a). The remaining mechanical properties for the intact rock matrix were derived from the literature (Colella et al. 2017; Croce et al. 2014; Evangelista et al. 2000), taking into account the Neapolitan Yellow Tuff formation, as it is the most similar to the PET (Table 1).

**Table 1:** Mechanical input parameters of the Punta Eolo Tuff (PET) referred to the rock matrix and to the discrete fracture network (DFN), and thermal input parameters of the PET used in the Irazu model

**Rock matrix mechanical properties**													
Triangular FEM-elements					Cohesive crack-elements								
Elastic properties					Strength properties						Stiffness properties		
Constitutive law	Density (γ_d_)	Young's modulus (*E*)	Poisson's ratio (*v*) *	Viscous damping coefficient (β)	Fracture model	Friction angle (ϕ) *	Cohesion (*c*)	Tensile strength (*σ*_t_)	Mode I fracture energy (*G*_I_)	Mode II fracture energy (G_II_)	Fracture penalty (*p*_f_)	Normal contact penalty (*p*_n_)	Tangential contact penalty (*p*_t_)
	kN/m^3^	[Pa]	[-]	[-]	[-]	[°]	[Pa]	[Pa]	[N/m]	[N/m]	[Pa]	[Pa۰m]	[Pa/m]
Plane Strain	1750	7.00E+08	0.3	1 (stat.) / 0.02 (dyn.)	Isotropic	30	1.25E+06	5.00E+05	10	100	7.00E+09	7.00E+09	1.00E+12
**Discrete fracture network properties**													
Strength properties										Stiffness properties			
Failure criterion	JRC	JCS	Basic friction angle (*ϕ*_*b*_)	Residual friction angle (*ϕ*_r_)	Barton friction agle (ϕ)	Cohesion (*c*)	Tensile strength (*f*_t_)	Mode I fracture energy (*G*_I_)	Mode II fracture energy (G_II_)	Fracture penalty (*p*_f_)	Normal contact penalty (*p*_n_)	Tangential contact penalty (*p*_t_)	
	[-]	[Pa]	[°]	[°]	[°]	[Pa]	[Pa]	[N/m]	[N/m]	[Pa]	[Pa۰m]	[Pa/m]	
Barton-bandis	8	5.00E+06	30	25	42	5.00E+03	2.50E+03	1	5	7.00E+09	7.00E+09	1.00E+12	
**Thermal properties**													
Conduction model	Thermal expansion model	Specific heat capacity (*C*_p_)	Thermal conductivity (*k*)	Linear thermal expansion coefficient (α)									
		J/(kgK)	W/(mK)	1/K									
Isotropic	Isotropic	880	2.5	1.00E-05									

*From Colella et al. (2017); Croce et al. (2014); Evangelista et al. (2000)

The fracture parameters were instead defined using empirical relationships derived from the literature and following the guidelines provided in the Irazu Theory Manual (Geomechanica Inc. 2024a) (see Supplementary Materials).

The modelling approach was designed to be flexible and goal-oriented, with the selection of domain configurations varying depending on the specific aim of each analysis. Rather than applying the same geomechanical setting across all simulations, the assumptions related to pre-existing joints were adapted to explore particular aspects of the sea cliff. Accordingly, three structural configurations were employed (Fig. 3a):

- TYPE I—intact rock: Used to evaluate the potential nucleation and growth of new fractures in an initially intact mass, allowing for a focused investigation of stress-driven damage evolution (e.g. basal notch development and undercutting).
- TYPE II—DFN rock mass: An embedded DFN is used to assess the mechanical response where the connectivity and orientation of pre-existing joints govern large-scale deformation and failure patterns. In particular, two sets of non-persistent joints were introduced to reproduce the high-angle fracture pattern observed at Punta Eolo. A gently counter-slope set (J0), dipping at 4°, represents the tuff layering, whereas a second, sub-vertical set (J1) replicates the parietal fractures mapped along the perimeter of the promontory. Equivalent Mohr-Coulomb strength parameters were evaluated for the rock joints by linearising the Barton (1973) criterion at an appropriate low stress level (Table 1). In particular, joint roughness coefficient (JRC) and basic friction angle (*ϕ*_*b*_) were estimated through in situ measurement according to ISRM (1978) guidelines. The JCS parameter was set equal to the UCS strength value since it is often considered inappropriate for soft rocks (Barton and Choubey 1977). As the DFN explicitly accounts for the presence of rock bridges through the geometrical persistence of discontinuities within the rock mass, joints were modelled as broken elements, with no cohesion or tensile strength assigned. In this framework, the mechanical contribution of rock bridges is provided by the intact rock between discontinuity segments, rather than by artificial cohesive parameters assigned to the joints.
- TYPE III—parietal joint model: A small number of sub-vertical joints near the cliff face, as observed in the field, are introduced to explore the influence of parietal discontinuities, i.e. joints that are likely to have a localised but potentially destabilising effect, especially in response to external factors. These are spaced 2 m apart (Fig. 1c), reflecting the typical arrangement observed in parietal secondary joint sets. Field surveys indicate that this set of discontinuities displays both scarp-slope and dip-slope orientations, with measured dips ranging within ± 5° from vertical. Based on these observations, joints were idealised as purely vertical (90°) in the numerical model, in order to capture their dominant mechanical behaviour while avoiding unnecessary geometrical complexity. Unlike the DFN-based configuration adopted in TYPE II, which statistically spatialises fracture systems within the rock mass, the TYPE III setup follows a deterministic approach aimed at explicitly reproducing the structural conditions in the immediate vicinity of the cliff face, where parietal joints exert a first-order control on local stability and damage localisation. In this configuration, joints were considered as cohesive or cohesionless (broken) joints depending on the aims of the analysis.

All FDEM simulations reported below employed elastic TFEs and an isotropic fracture criterion for the CCEs.

## Modelling geostructural control

**Fig. 4:**
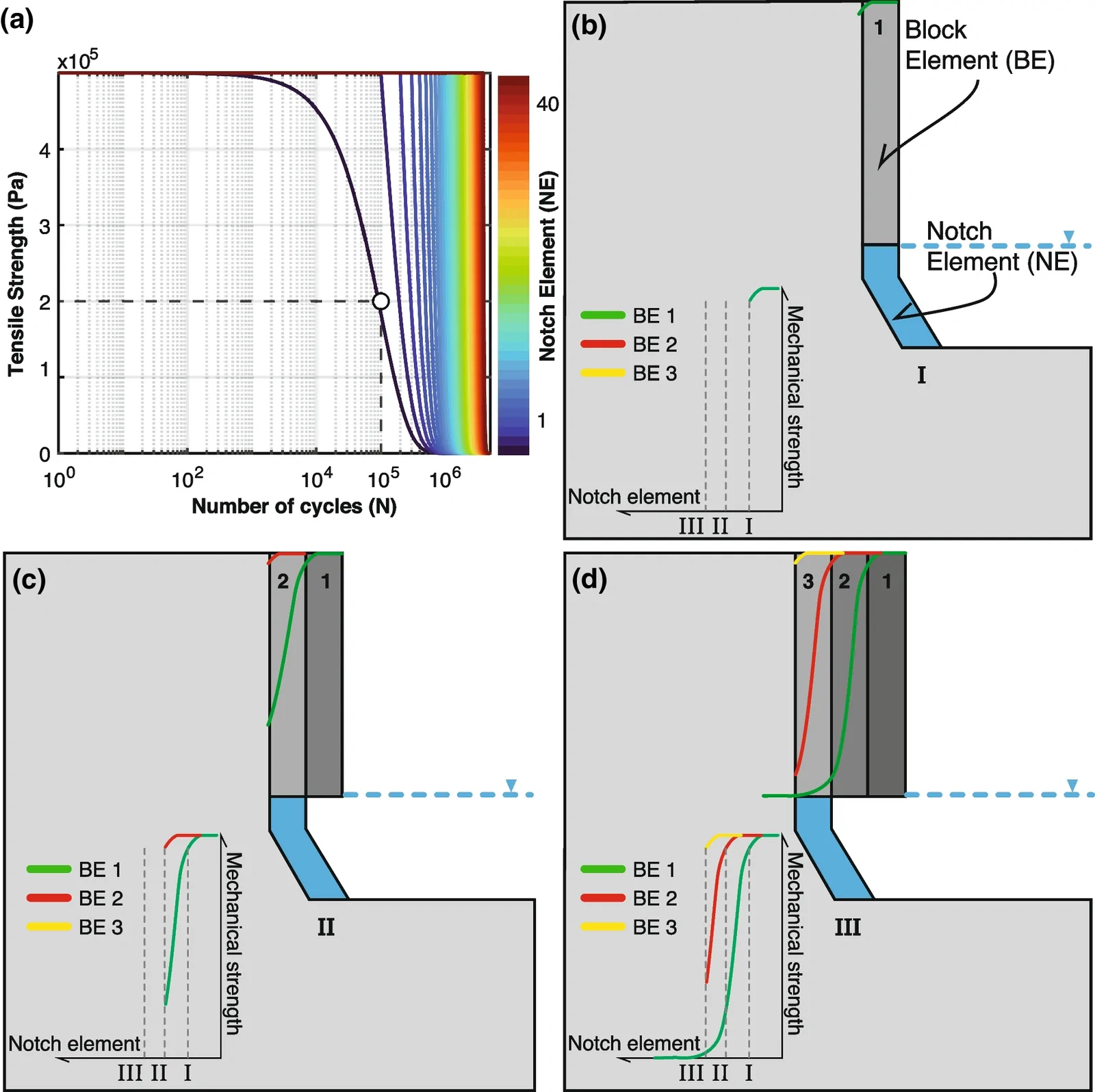
**a)** Example of S–N curves applied to the tensile strength parameter for all forty block and notch elements (NEs) considered in the simulations. As an example, the curves illustrate the progressive tensile strength decay as a function of the number of loading cycles (*N*). The white dot indicates the characteristic number of cycles (*N*_0_ = 100k) adopted in this study. **b**–**d)** Conceptual illustration of the coupling between basal notch element (NE) erosion and strength decay of the block elements (BEs). The blue area indicates the newly excavated NE, while the overlying BEs (in grey) progressively experience mechanical strength degradation. The inset diagrams report a simplified S–N curve representing the corresponding decay of mechanical parameters with increasing notch erosion at each panel. The decay of each BE begins only when the underlying NE starts to be excavated, thus ensuring a realistic coupling between the geomorphic evolution of the basal notch and the weathering-induced weakening of the overlying rock mass

A FDEM model was developed to understand the role of basal notch formation in the cliff stability of Punta Eolo. Notch development was modelled in the FDEM simulation using a series of incremental excavations. Each notch element (hereafter “NE”) was assigned a constant geometry corresponding to the entire portion of the cliff below mean sea level (Fig. 4). This approach was adopted to (i) minimise the influence of progressive changes in notch geometry as the depth of the undercut within the cliff increases, and (ii) ensure the reproducibility of the cliff response to each successive NE excavation undercutting step.

The relationship proposed by Sunamura (1992) was applied by inputting site-specific parameters for Punta Eolo, including rock resistance and a mean wave height of 0.2 m derived from the VFL available data. Based on these drivers, a notch retreat rate of 0.05 m/year was estimated for the cliff. Because this retreat rate is smaller than the minimum mesh size (0.1 m), a notch thickness of 0.25 m was implemented for each excavation step, equivalent to approximately 5 years of erosion. Notch excavation was implemented using the core modulus reduction method (Geomechanica Inc. 2024b), whereby the Young’s modulus of the NE was gradually reduced before deletion after 100,000 mechanical steps, ensuring equilibrium after each stage. The notch retreat process was analysed by considering both TYPE I (intact) and TYPE II (DFN) cliff domain setups.

Some additional details on the use of Sunamura’s (1992) relationship report in this study are provided in the Supplementary Material.

## Modelling the sea waves’ impact on the cliff

### Hydrodynamic modelling

The mesh-based CFD solver OpenFOAM v2406, whose applicability to modelling wave-structure interactions has been demonstrated in previous studies (Sobhani et al. 2026), was employed to capture the local impacts of a wave on the vertical cliff face of Punta Eolo.

The CFD model configuration is designed with sufficient spatial extent to allow wave generation, propagation, impact, and reflection from the cliff without interfering with the boundary conditions or making the simulation time suboptimal. The geometrical characteristics of the domain were inferred from the analysis of the available digital terrain models (DTMs) derived from aerial and bathymetric surveys performed in the area of Punta Eolo. These DTMs were processed in MATLAB using the TopoToolbox program (Schwanghart and Scherler 2014) to provide a realistic representation of the cliff and seafloor morphology. The terrain analyses proved how a vertical cliff can be assumed as representative of the morphological conditions of the Punta Eolo study area, where mean water depths range from 4 m increasing along the sloping seabed to 8 m at a horizontal distance of 50 m offshore (Fig. 3b). Beyond this slope and consistent with the bathymetry data, the seabed depth remains constant at 8 m for an additional 100 m. The CFD domain reflects the average bathymetry in the area with a vertical cliff extending 20 m above the still water level (SWL) (Fig. 3b). The computational mesh was divided into five regions, each characterised by a different resolution (Fig. 3b). This zoning strategy ensured numerical accuracy while minimising computational cost. Mesh refinement was concentrated in areas where hydrodynamic interactions are most intense and sensitive to grid resolution. In particular, for region 5, located in front of the cliff, the cell height was set to 0.05 m with the aim of sharing the same spatial resolution with the FDEM mesh used to discretise the cliff face.

The CFD simulations considered two representative cases: solitary and periodic waves. The solitary-wave simulations were included because they provide a physically consistent representation of extreme long-wave events and therefore offer upper-bound estimates of wave-induced pressures. Conversely, periodic waves were also simulated to better represent typical sea states and supply impulsive impacts that are more representative of real waves approaching natural coasts. Since the goal of this study is to evaluate the mechanical response of the cliff under energetic wave loading, the simulations adopted a representative sea wave height of 2 m, corresponding to rough-sea states affecting the study area, with a wave period of 4 s. This choice is justified by available observations at Punta Eolo, where fair-weather conditions typically involve significant wave heights of 0.2 m, but energetic events with heights up to 2 m have been recorded (Fig. 1d). Smaller wave heights (e.g. 0.2 m) generate pressure fluctuations that are too low to be reliably resolved within the field-scale FDEM domain, where numerical noise and mesh discretisation limit sensitivity to low-amplitude pressure variations. Therefore, adopting a 2-m wave height ensures both physical relevance—capturing rough-sea conditions—and numerical robustness in resolving wave-induced stress perturbations within the coastal cliff.

Some additional details of the CFD principles and numerical model setup are provided in the Supplementary Material.

## Modelling the thermal forcing effect

A FDEM model was developed for the Punta Eolo cliff to analyse how thermal fluctuations may affect the mechanical stability of the coastal rock mass. The TYPE III (parietal joints) configuration was selected to isolate the effects of thermal cycling on vertical, wall-parallel joints, which are typical of the cliff sector. This simplified configuration provided greater control over the simulation process and allowed for a more direct assessment of the influence of temperature variations on joint behaviour. The joints were treated as cohesive so that the potential for thermally induced joint failure could be explicitly assessed.

Thermal propagation into the cliff face was modelled using the 1D semi-infinite solid solution of Carslaw and Jaeger (1959), which allows for the quantitative evaluation of the typical penetration depth (TPD) and of the thermal active layer (TAL). The TPD represents the distance to which a thermal front can propagate within the solid medium, and can be interpreted as a theoretical parameter that quantifies the characteristic length scale of the exponential attenuation of thermal oscillations. The portion of the rock mass where temperature variations remain appreciable over time is commonly referred to as the TAL (Gischig et al. 2011; Marmoni et al. 2020). Its thickness corresponds to the part of the medium effectively influenced by periodic thermal fluctuations and typically spans multiple TPDs, depending on the temperature threshold considered. In the present study, no specific threshold was imposed; thus, the TAL in this case defines the entire region experiencing measurable temperature variations.

λ and ${C}_{p}$ were determined through differential scanning calorimetry (DSC) and modulated differential scanning calorimetry (MDSC) laboratory tests carried out by Certimac s.r.l (Table 1). The linear thermal expansion coefficient (α) was taken from the Neapolitan Yellow Tuff literature (Aversa and Evangelista 1993; Heap et al. 2018b; Rosato et al. 2024), the closest analogue to the PET.

To capture both horizontal and vertical thermal propagation, temperatures were monitored along three profiles: (i) a horizontal line 10 m below the upper model boundary (ML1); (ii) a vertical line 10 m from the cliff face (ML2); and (iii) an inclined 45° line from the cliff edge (ML3).

Following Marmoni et al. (2020), the thermal input was designed as an equivalent signal representing both daily and seasonal rock temperature cycles recorded at Punta Eolo. Because the VFL monitoring period was short to date, long-term air temperature records from iLMeteo (https://www.ilmeteo.it/portale/archivio-meteo/Ventotene) covering the past 10 years were analysed to obtain a more statistically robust dataset. Daily and monthly means and thermal amplitudes were calculated for the iLMeteo and VFL datasets and applied consistently to the available rock temperature records. The processed air and rock temperature series were then compared to derive empirical conversion relationships, which were used to reconstruct rock-temperature histories for periods when only air-temperature data were available. To test the model’s sensitivity to thermal forcing, additional scenarios were simulated in which annual temperature amplitudes were increased by + 10 °C and + 20 °C relative to the reference case. Moreover, to make results transferable, the thermomechanical analyses considered a high-conductivity and more thermally responsive medium, characterised by a ${C}_{p}$ of 880 J/kgK, a λ of 2.5 W/mK, and an α of 1۰10^−5^ 1/K. Although these properties differ from those typical of tuffaceous lithologies, they are consistent with other rock types, such as granites and basalts (Carmichael 1982; Robertson 1988; Steiger et al. 2010; Zhu et al. 2022), where steep cliffs are also common.

The daily thermal cycle was applied over 30 days to ensure numerical stabilisation of the short-period thermomechanical response, allowing a steady cyclic regime rather than a single transient oscillation. Likewise, the seasonal thermal signal was imposed over a 3-year period to stabilise the long-period thermal field, ensuring a realistic and stable initial state before the subsequent long-term simulations.

Thermal boundary conditions were uniformly applied to both the cliff face and upper portion of the model (Fig. 3), while a constant temperature of 22 °C was prescribed below sea level. This value represents the annual average water temperature recorded at the site by the AWAC sensor, with seasonal fluctuations ranging from a minimum of 14 °C in winter to a maximum of 30 °C in summer. in accordance with average water temperatures recorded at the site by the AWAC sensor.

The periodic thermal signal was discretised with a resolution of 300 s, and simulations were initialised at 21 °C, corresponding to the processed annual mean rock temperature.

The TM modelling was carried out by coupling each thermal step with a sufficient number of mechanical time steps to re-establish quasi-static equilibrium.

Some additional details of the Carslaw and Jaeger (1959) and of the graphical representation of the thermal inputs used are provided in the Supplementary Material.

## Dynamic FDEM modelling

CFD-derived pressure field results were used as dynamic boundary conditions for the FDEM simulations. Wave impacts were simulated using both the TYPE I (intact) and TYPE III (parietal joints) domain configurations (Fig. 3a). The TYPE 3 setup, in which near‐face joints are explicitly represented as open discontinuities, was designed to investigate how pre-existing jointing influences wave impact on the cliff face, providing a more targeted assessment of fracture-controlled deformation near the cliff face.

To prevent artificial wave reflections during the dynamic simulation of wave impact, the FDEM domain was extended while preserving the geometric and meshing characteristics of the cliff zone. In particular, the refined mesh along the cliff face was kept identical to that used in the other simulations to ensure consistency. Initially, the model was brought to elastic and elasto-plastic equilibrium using a viscous damping coefficient (*β*) set to 1. Subsequently, the *β* coefficient was reduced to 0.02 to account for wave energy dissipation. This value is consistent with the seismic quality factor (*Q*) for tuffaceous lithologies (e.g. Carroll 1994; Serlenga et al. 2016; Vanorio et al. 2002).

Sea wave loading was applied in the form of time-dependent pressures derived directly from the CFD simulations. Because both the CFD and FDEM meshes share the same spatial resolution (0.05 m), the computed pressures could be directly transferred to the corresponding FDEM elements without interpolation or scaling. These impact pressures were applied to the cliff face above mean sea level according to the simulated wave runup. Below sea level, hydrostatic pressure was maintained to preserve the pre-equilibrium as dynamic wave pressures below sea level are negligible. To isolate the dynamic response induced by wave impact, pre-impact background fields (stress, velocity, displacement) were filtered during post-processing using ParaView (Ahrens et al. 2005).

The dynamic cliff response was investigated at different control points (Fig. 9a) to capture both horizontal and vertical propagation of wave-induced disturbances, and to detect differences in the cliff deformation response between TYPE I (intact) and TYPE III (parietal joints) setups.

## Modelling fatigue-driven degradation under cyclical actions

Block elements (BEs) were introduced to represent the portions of the parietal rock mass subjected to progressive mechanical degradation induced by marine-driven weathering processes, which preferentially affect exposed cliff faces (Furlani et al. 2014) (Fig. 4b–d). To provide a simplified yet physically consistent coupling between mechanical property decay and basal undercutting, the spatial extent and temporal activation of the BEs were linked to the sequential excavation of the underlying notch elements (NEs). Accordingly, the mechanical weakening of each BE was activated only when excavation of the corresponding underlying NE began, ensuring a controlled interaction between notch development and strength degradation. The BEs were discretised with the same thickness as the underlying NEs (0.25 m).

The mechanical degradation of the BEs was introduced in the FDEM model through a cyclic-fatigue formulation based on the S–N approach (Fig. 4a), which describes the exponential decay of material strength (*S*) with respect to the increasing number of loading cycles (*N*) (Brighenti 1979). To this end, the simplified exponential relation proposed by Jafari et al. (2003), later applied to coastal cliffs by Adams et al. (2005), was adopted: 

1 $${\tau}_{f}= {\tau}_{p}{e}^{\frac{-N}{N0}}{e}^{\frac{-\tau c}{\tau p}}$$

where ${\tau}_{p}$ is the peak (i.e. unaltered) rock failure strength, *N* is the number of loading cycles experienced by the sea cliff, *N*_0_ is a characteristic fatigue parameter controlling the rate of degradation (i.e. the number of cycles necessary to reduce the rock mass strength by 1/*e* of its original value), and ${\tau}_{c}$ is the maximum stress experienced during a loading cycle. Because ${\tau}_{c}$ induced by wave impact on sea cliffs is small relative to intact rock strength, the second exponential of Eq. 1 is negligible and degradation is controlled primarily by the accumulated number of cycles (Adams et al. 2005).

For the present research, *N* represents the combined number of stress cycles induced by the environmental stressors acting simultaneously on the cliff (e.g. thermal cycles, wetting/drying cycles, wave impacts). Rather than resolving individual cyclic actions explicitly, *N* is treated as a normalised measure of integrated fatigue damage. The characteristic cycle number *N*_0_ was set to 100,000 mechanical steps, calibrated to match the time required to excavate one NE (approximately 5 years) based on Sunamura’s erosion retreat model for the Ventotene coastal environment. This implicitly assumes (i) that weathering-induced degradation of the cliff face occurs at a rate commensurate with basal undercutting, given their shared exposure to marine influence; and (ii) the 100,000 cycles represent not a literal count of individual events, but rather a normalised metric that summarises the integrated effect of multiple cyclic processes, each operating at different frequencies but contributing collectively to rock mass fatigue.

Starting from the peak strength ${\tau}_{p}$, the mechanical parameters of each BE were progressively reduced with increasing loading cycles, following Eq. 1. Strength degradation was implemented in correspondence with the geomorphological evolution of the notch: for each BE, the onset of weakening was activated only when excavation of the underlying NE began, thus ensuring a realistic coupling between basal undercutting and progressive loss of rock strength around the notch.

The S–N law was applied to the key mechanical parameters affected by cyclic weakening, namely cohesion, friction angle, and tensile strength (for CCEs), and Young’s modulus (for TFEs). These parameters are consistent with experimental evidence (Cerfontaine and Collin 2018; Liu and Dai 2021), and slope stability analyses of progressive rock slope failure (e.g. Eberhardt et al. 2004; Stead and Eberhardt 2013; Styles 2009). Parameters were progressively reduced from peak to minimum threshold values selected to preserve numerical stability and prevent non-physical collapse of the modelled domain. In particular, the minimum thresholds were set equal to 5° for the friction angle (*ϕ*), 125 kPa for the cohesion (*c*), 50 kPa for the tensile strength (*σ*_t_), and 70 MPa for the Young’s modulus (*E*). These minimum thresholds were defined as lower-bound numerical constraints to ensure solver stability during the advanced stages of the fatigue process, representing a state of complete mechanical degradation.

## Results

### Geo-structural control on cliff failure mechanism

**Fig. 5:**
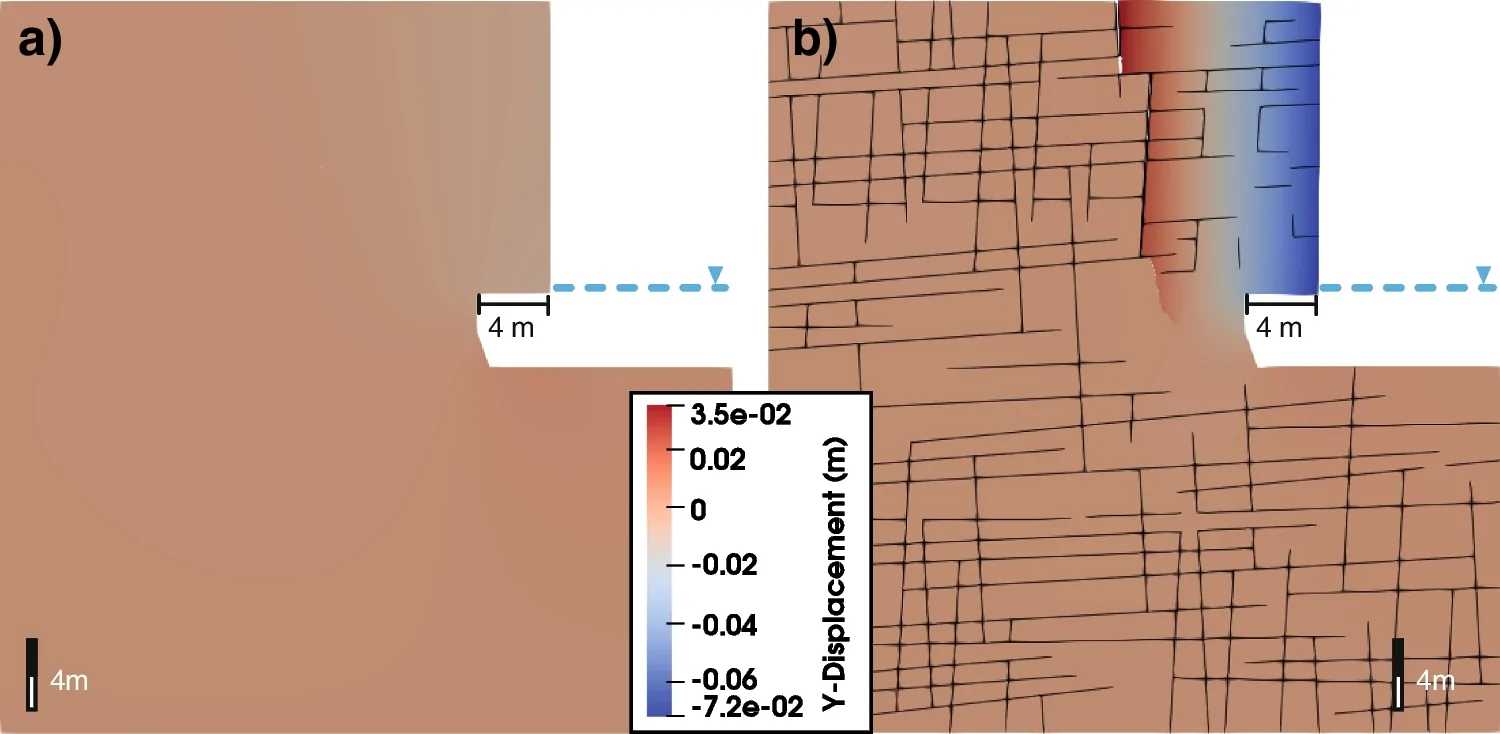
Vertical displacement field (Y-displacement) obtained from the simplified-geometry model under the assumption of **a**) absence (TYPE I setup) and **b**) presence of a DFN (TYPE II setup). In the TYPE I setup, the excavation of several notch elements (NEs) does not produce significant effects, whereas in the TYPE II setup the excavation of a 4-m-wide notch induces vertical displacement concentration on a parietal block, promoting DFN failure and joint opening

The excavation of 40 NEs, corresponding to a total basal undercutting of 10 m, was insufficient to induce any destabilising effect in the TYPE I (intact) cliff setup. In particular, the elasto-plastic equilibrium condition remained stable throughout the entire simulation, preventing any strain localisation, as well as any fracture initiation and cliff instability (Fig. 5a). On the contrary, the geomechanical setting represented in the TYPE II model, which incorporates a DFN, induced a markedly different response to the notch excavation (Fig. 5b). In this case, the rock mass reacts to the simulated undercutting process through the propagation of pre-existing fractures, the formation of new discontinuities, and ultimately through the collapse of the frontal portion of the cliff face. Specifically, once the notch reaches a depth of approximately 4 m, the displacement field generated in the model becomes concentrated within a large rock block approximately 11 m in length. From this point onward, pre-existing fractures within the rock mass tend to open progressively, failing under mode I, leading to the initiation and propagation of new fractures emanating from the tips of these existing discontinuities. This process results in the mobilisation of the aforementioned block, which undergoes a toppling failure mechanism.

### Thermally induced effect

Thermal simulations using the properties of PET show that heat transfer is limited to shallow depths, with daily and annual TPDs of approximately 0.1 m and 2 m, respectively (see Supplementary Materials), consistent with Carslaw and Jaeger (1959). Under these conditions, thermal forcing remains mechanically ineffective; no strain softening or fracture initiation is observed, even when temperature amplitudes are artificially increased by up to + 20 °C. These findings indicate that PET thermal properties do not promote joint damage under current or intensified thermal regimes.

**Fig. 6:**
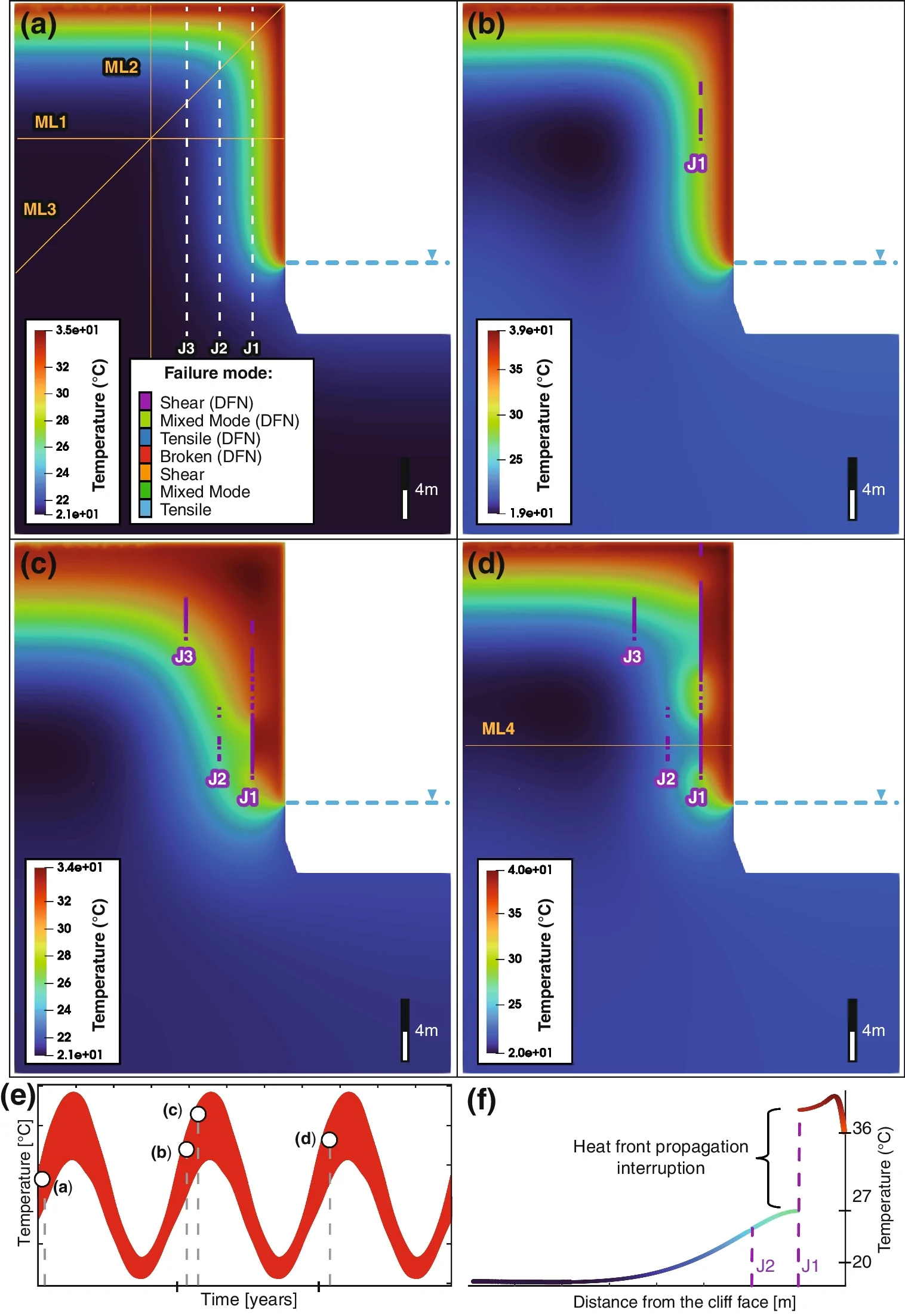
Isothermal maps produced under an annual thermal input with a + 20 °C excursion relative to the realistic case, for a material parametrised with higher thermal conductivity and thermal expansivity than the PET. The results show the progressive failure of pre-existing joints (see legend in panel **a**). Panels **a**–**d** display the thermal fields at different stages of the simulation, as indicated in panel **d** showing the applied composite thermal input: **a** start of the simulation. Monitoring lines (ML) and joints’ traces (Jn) are plotted, but no failure occurs; **b** beginning of the warm season in the second year; **c** warm season of the second year; **d** beginning of the warm season in the third year; **e** schematic representation of the 3-year composite annual thermal input used in the simulation, with markers indicating the stages illustrated in panels (a–d); **f** temperature profile along the ML4 monitoring line, highlighting the interruption of heat-front propagation at the location of the broken joint J1

**Fig. 7:**
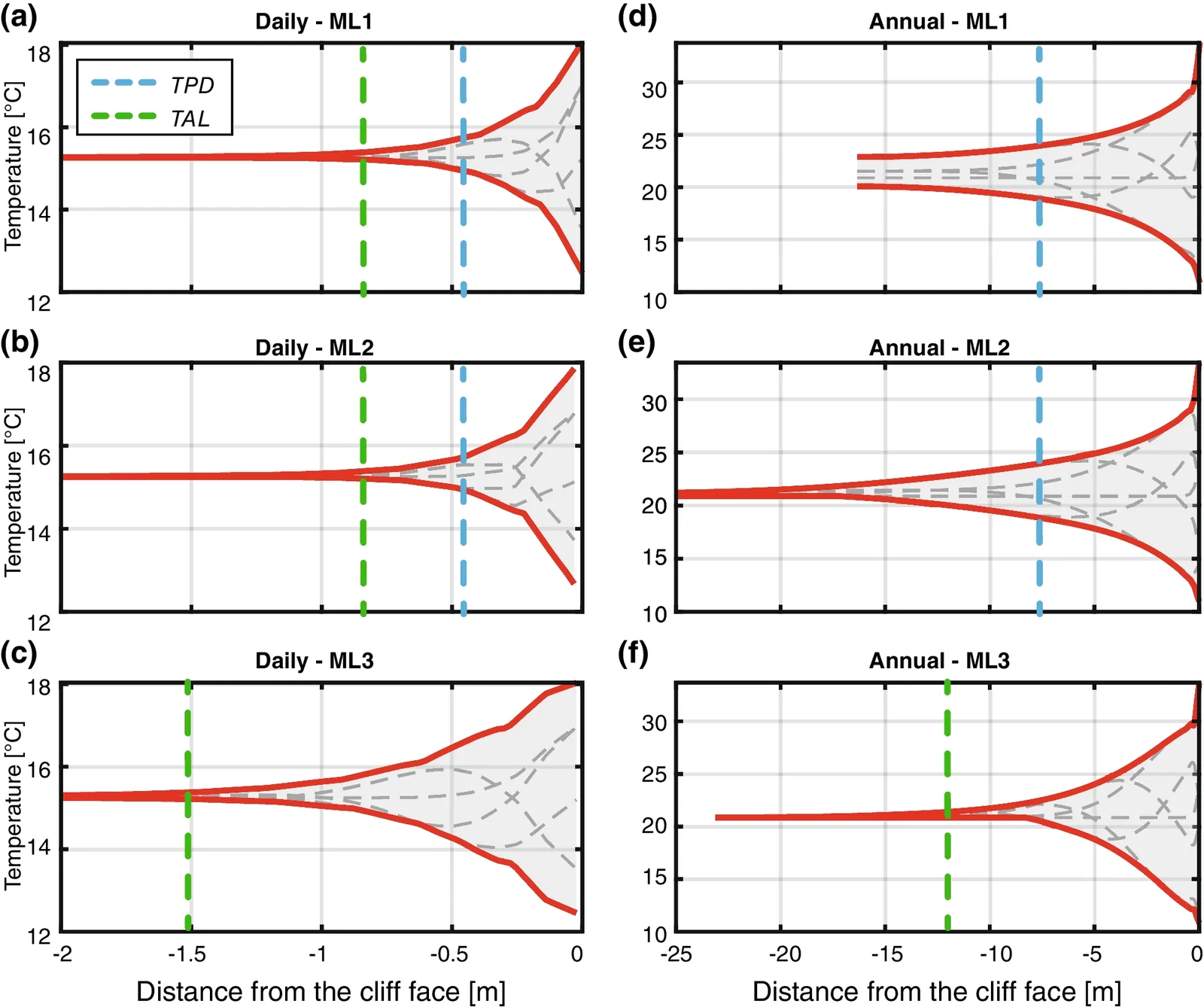
Envelopes of temperature time series monitored along ML1, ML2, and ML3 (locations in Fig. 6) under daily (**a**–**c**) and annual (**d**–**f**) composite thermal inputs applied to a rock mass parametrised with thermal properties different from those of the Ventotene tuffs. Red curves mark the portions of the rock mass affected by the imposed temperature excursions, while grey-shaded areas with dashed lines represent intermediate monitoring lines recorded during the simulation. The typical penetration depth (TPD) and the thermally active layer (TAL) are indicated by blue and green dashed lines, respectively. The TPD is not shown along ML3 due to constructive interference between heat fronts, which violates the 1D assumption of the Carslaw and Jaeger (1959) analytical model. Conversely, the TAL is not reported along ML1 and ML2 in the annual simulations (panels **d**–**e**) because these monitoring lines fall entirely within the thermally active zone

In contrast, simulations using more conductive and expansive rock properties, coupled with amplified annual excursions, show a markedly different response (Fig. 6). In this scenario, higher thermal efficiency leads to a TPD of up to 7.5 m (ML1 and ML2 in Fig. 7), with the fringe area (ML3 in Fig. 7) exhibiting even deeper perturbations due to constructive interference between the crest and cliff face heat fronts. This localised deepening results in a TAL of approximately 15 m, which, unlike the PET case, fully overlaps with the discontinuity network (J1–J3).

Under a + 20 °C annual amplitude increase, this overlap triggers progressive joint failure. Fracturing initiates as mode II shear failure at the upper tip of J1 and propagates downward to J2 and J3 during subsequent warming phases (Fig. 6b–e).

Notably, joint failure modifies the thermal field: the fractured surfaces act as thermal barriers, significantly slowing conduction. This is evidenced by a temperature drop from 38 to 27 °C across the broken surfaces of J2 (Fig. 6f), highlighting the strong coupling between mechanical damage and thermal propagation.

### Dynamic response to marine forcing

**Fig. 8:**
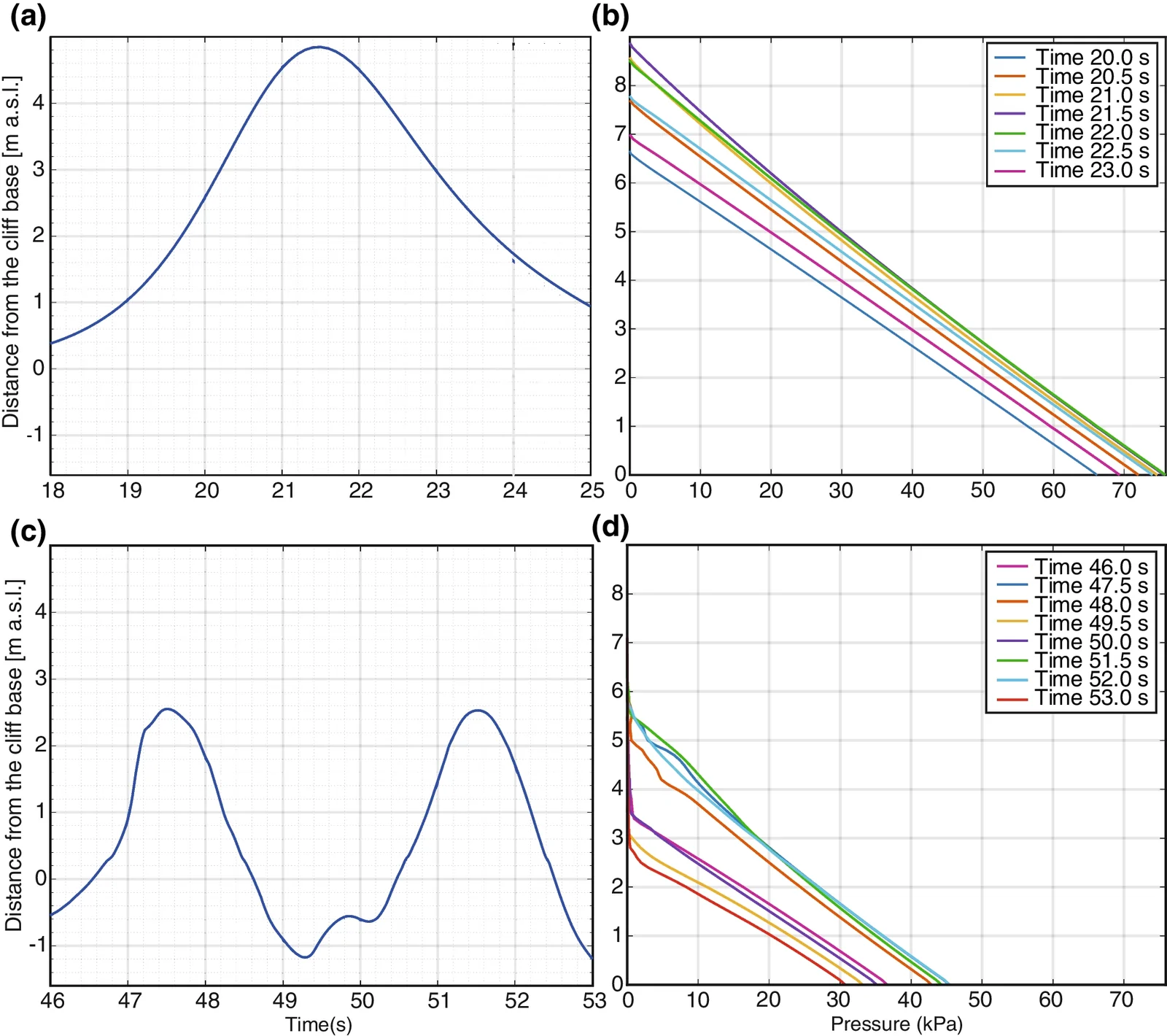
**a**) Runup time history on the cliff under a 2-m solitary wave from 18 to 25 s. **b**) Pressure distribution on the cliff under a 2.0-m-high solitary wave from 20 to 23 s. **c**) Runup time history on the cliff under a 2-m-high periodic wave from 46 to 53 s. **d**) Pressure distribution on the cliff under a 2.0-m-high periodic wave from 46 to 53

CFD analyses show that hydrodynamic pressures consistently concentrate around the SWL for both solitary and periodic waves, with a linear decay toward the cliff crest (Fig. 8).

For the solitary wave scenario, the maximum wave runup (WRL) reaches approximately 4.8 m, generating a peak pressure of roughly 40 kPa at the SWL (Fig. 8a, b). In this case, the pressure distribution follows a triangular profile that expands upward as the cliff face becomes inundated, with the maximum force coinciding with the peak runup.

Conversely, periodic waves exhibit a lower WRL (about 2.5 m) and a significantly reduced peak pressures at the SWL (≈10 kPa), approximately one-quarter of the solitary-wave value (Fig. 8c, d). While the pressure distribution remains generally triangular, transient irregularities occur due to wave breaking and splash-up effects. Despite the lower pressures, periodic waves are characterised by a highly impulsive nature, with a very short rise time (*t*_rise_ < 0.5 s) that necessitates a dynamic rather than purely static treatment of the cliff’s mechanical response.

Because the applied hydrodynamic pressures are very small (i.e. < 100 kPa) relative to typical geomechanical stress levels, resolving the full CFD-derived pressure time history in the FDEM model would be computationally inefficient and numerically unreliable. Instead, emphasis was placed on accurately capturing the peak-impact event associated with the peak pressures. Although this represents a simplification of the full dynamic loading history, it is appropriate given that Irazu currently lacks dynamic boundary conditions (e.g. free-field margins) required to stably apply multi-second transient loads. Importantly, the physical process of interest is the impulsive wave impact, which is characterised by a very short *t*_rise_ on the order of milliseconds (Griffiths 1993). Therefore, the triangular pressure distribution was assumed to act almost instantaneously (*t*_rise_ = 0.005 s), thus replicating an impulsive wave impact.

**Fig. 9:**
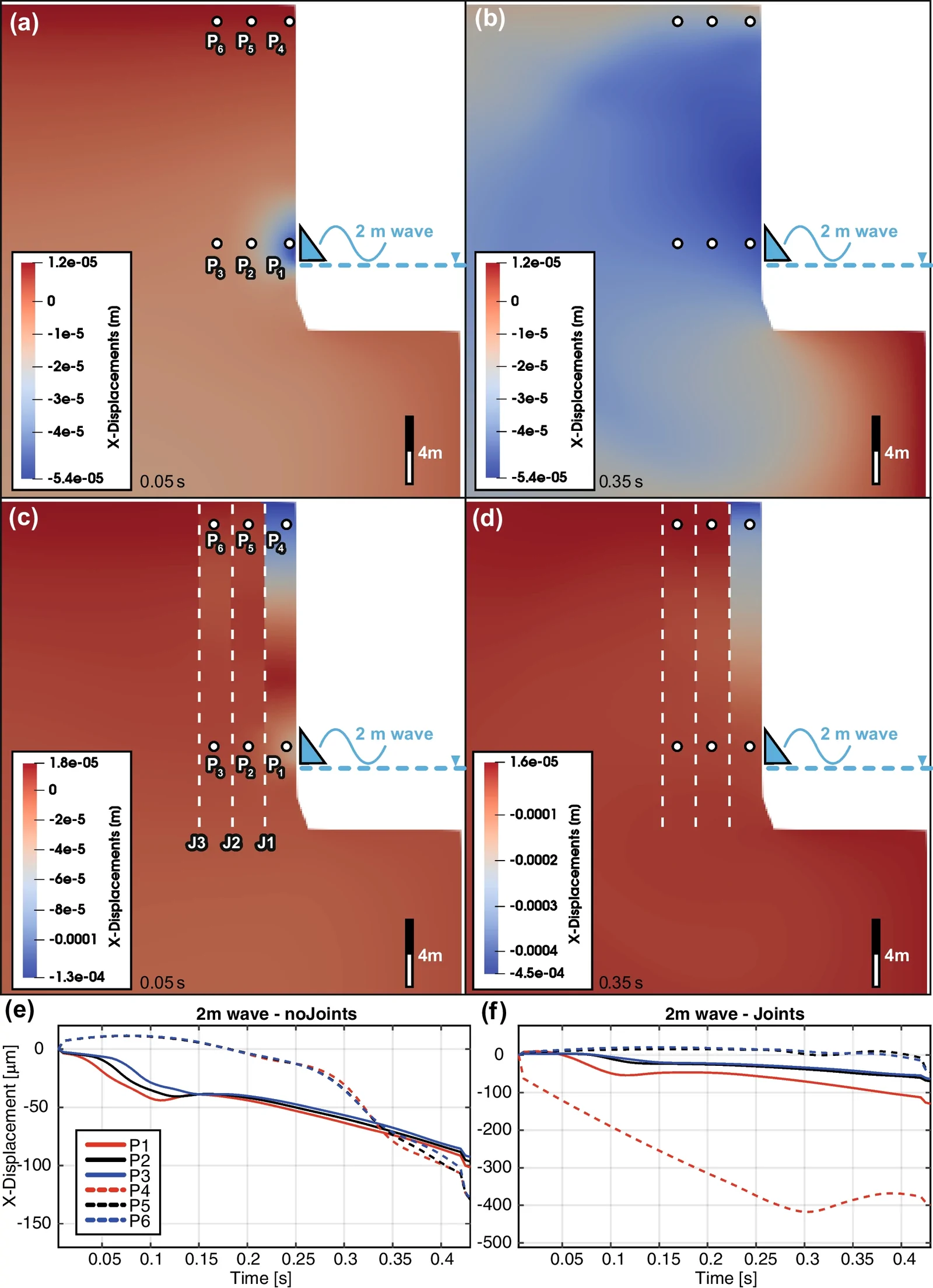
Results of the FDEM dynamic analysis for the 2-m-high wave. **a)** Response at 0.05 s and **b)** without vertical joints (TYPE I setup). **c)** Response at 0.05 s and **d** with three vertical joints (TYPE III setup). Panels **e)** and **f)** show the time series of horizontal displacements recorded at monitoring points P1–P6 for the 2-m-high wave under TYPE I (**e**) and TYPE III (**f**) configurations

In the intact model (TYPE I), wave propagation is primarily controlled by material damping (*β* coefficient), resulting in a radially symmetric displacement field (Fig. 9a, b). The impact induces inward-directed (negative) displacements that decay monotonically with distance from the impact zone (Fig. 9e). As the wave front reaches the cliff crest, the propagation geometry transitions from horizontal to vertical, causing a nearly synchronous response at the upper monitoring points (P4–P6). There is then a phase of adjustment in the displacements associated with destructive interference of the reflected and transmitted wave fronts.

In contrast, jointed models (TYPES II and III) exhibit a perturbed propagation pattern due to the scattering and reflection of waves at joint boundaries (Fig. 9c, d). The outermost fracture (J1) partially intercepts and redirects the incoming stress wave, focusing energy within the parietal rock block. This leads to strong constructive interference: at P1, horizontal displacements reach values significantly higher than in the intact case (up to −180 mm), while the joint network simultaneously delays and attenuates wave transmission toward the cliff interior (Fig. 9f). Notably, reflections at the crest generate outward-directed (positive) displacements toward the sea, favouring an overturning moment.

Throughout the simulation, strain levels remain within the elastic regime. In particular, the applied wave-induced pressures generate displacements that are too small to activate strain softening in the CCEs of the mesh. Consequently, no new fracture initiation occurs in the absence of pre-existing or progressively accumulated rock mass damage.

### Progressive rock failure under cyclic stressors

The results obtained from the fatigue-driven degradation analysis were analysed with particular attention to the yielding state of the CCEs, as well as to the failure mode and fracturing pattern developed in the rock mass.

**Fig. 10:**
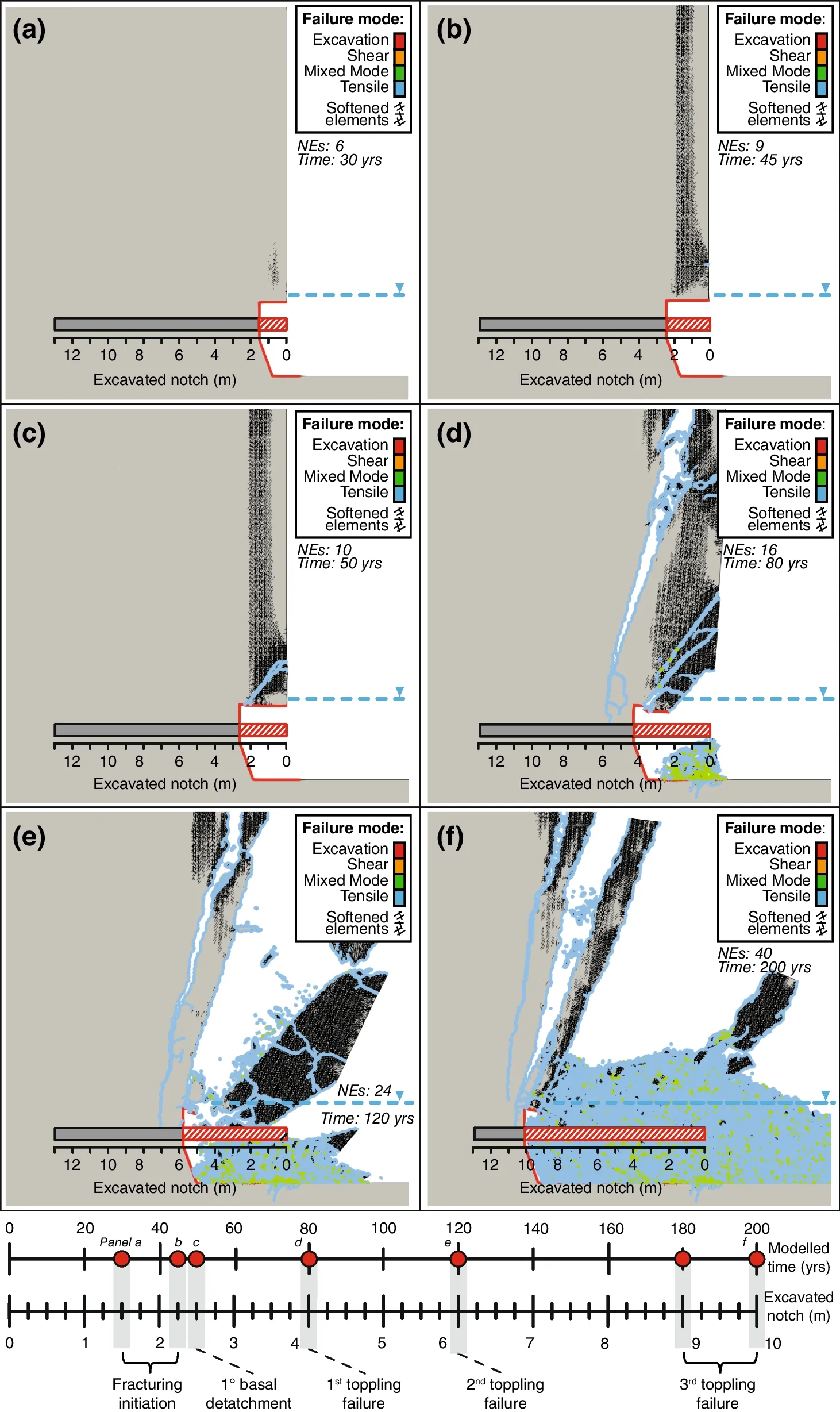
Evolution of the fracturing and failure processes during notch excavation obtained by decreasing the friction angle, cohesion, tensile strength, and Young’s modulus. Panels **a**–**f** illustrate the progressive development of softened cohesive crack elements (CCEs) and fractures, classified according to failure mode, as the notch excavation, represented by the red horizontal bar, progresses. The number of excavated notch elements (NEs) and the equivalent modelled time are reported under the legend of each panel. At the bottom of the figure, a synthetic timeline is provided, allowing the temporal evolution of the simulation to be directly related to the cumulative excavated notch depth. The red dots along the timeline indicate the key fracturing and failure events discussed in the text and illustrated in panels **a**–**f**

The first evidence of strain softening is observed between the excavation of the sixth and seventh NE, corresponding to 1.5–1.75 m of basal erosion, or about 30–35 years of retreat (Fig. 10a). At this stage, CCEs immediately above the first excavated NE begin to yield, marking the onset of progressive damage at the cliff base. With continued undercutting, the softening process intensifies, with critical conditions expanding both inland and upward (Fig. 10b). At this stage, its horizontal and vertical propagation are strongly influenced by the BE geometry and meshing, which promotes preferential upward growth of yielded CCEs within individual BEs rather than laterally spreading between adjacent blocks. The first evidence of macroscopic fracturing appears once the tenth NE is excavated (~ 2.5 m of basal erosion, or about 50 years), when a tension crack initiates and propagates (Fig. 10c). This extends from the cliff face to approximately 1.8 m above the sea level, where the BEs have already undergone more pronounced mechanical strength degradation. The crack follows a 45° downward inclined trajectory, isolating a 1.8-m-wide rock block that detaches by rockfall. Once the falling block reaches the seafloor, it fragments upon impact (Fig. 10d). Although the fragmentation process and particle size of the produced debris were not analysed here, the continued decay of mechanical properties within the detached block, and the decision not to remove the debris from the domain for computational efficiency, may lead to unrealistically high fragmentation upon impact. The detachment of this first small-volume block also creates a basal overhang, altering the local cliff geometry.

Subsequent fracture initiation occurs between 2.5 and 4.0 m of basal erosion (50–80 years) (Fig. 10d), when a much larger rock block approximately 3.5 m wide spanning the full cliff height detaches by toppling. When referenced to the cliff face after the first failure, this corresponds to an effective undercutting of about 1.95 m, indicating progressive amplification of instability driven by notch growth and near-face strength degradation.

The fracturing process is consistently accompanied by the softening of the CCEs within the NEs, which undergo a progressive loss of mechanical strength. In particular, softened CCEs coalesced into vertically continuous damage zones across the cliff, providing the necessary mechanical conditions for fracture nucleation to initiate from the upper cliff region. Sub-vertical sequences of fractures with counter-slope inclination of about 60 to 80° thus fail mainly in mode I and secondarily in shear and mixed modes (Fig. 10d, e).

As the simulation progresses toward its final stages, the fracturing process forms isolated rock blocks characterised by a distinctive columnar geometry approximately 2 m wide that spans the full height of the cliff (Fig. 10e). These later detach from the cliff through toppling as the simulation progresses toward its final stages (Fig. 10e, f). Overall, the fracture pattern that is generated induces an overhanging cliff geometry once the rock blocks detach.

## Discussion

**Fig. 11:**
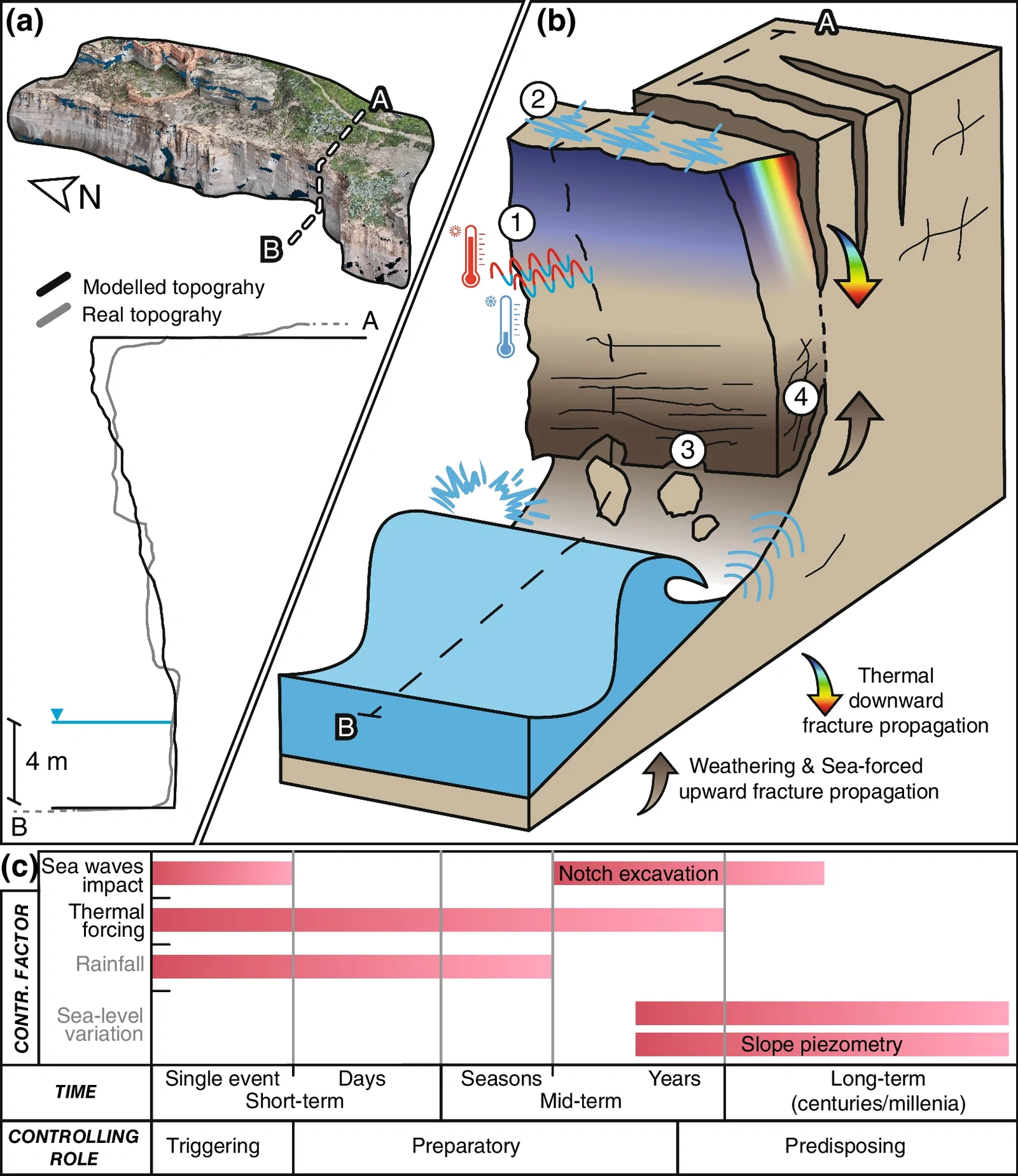
**a** Comparison between a real topographic cross-section of Punta Eolo cliff and the modelled one presented in Fig. 10f. **b** Conceptual sketch illustrating the main controlling factors affecting soft rocky coastal cliffs, such as those characterising the Ventotene coastline. The sketch highlights both thermally induced downward fracture propagation and weathering- and sea-forced upward fracture propagation. The numbered labels indicate (1) thermal forcing; (2) sea-wave-induced shaking; (3) notch excavation driven by repeated wave impacts; and (4) rock weathering processes acting along the cliff face. **c** Schematic representation of the controlling factors considered in this study and their associated temporal scales. The different forcings act over distinct time windows, ranging from short-term meteorological events (seconds to days), through seasonal to mid-term processes, up to long-term climatic and sea-level trends (centuries to millennia). Rainfall and sea-level variations are shown as examples (indicated by faded labels), illustrating additional controlling factors that could be incorporated into analyses similar to those performed in this research

The FDEM simulations demonstrate that the DFN is the primary predisposing factor for cliff failure at Ventotene. Gravity loading alone, or even basal notches exceeding 4 m in intact rock (TYPE I), fails to induce collapse (Fig. 5), indicating that structural predisposition must be coupled with weathering to drive instability. While undercutting in DFN-integrated domains produces toppling failures consistent with observations (Fig. 11a), the required notch dimensions are irrealistic and larger than those found in situ. This suggests that marine erosion should be interpreted primarily as a preparatory process that progressively destabilises the rock mass rather than a sole driver of instantaneous failure.

Meteo-climatic forcings exhibit a clear hierarchy in mechanical effectiveness. Coupled CFD–FDEM simulations show that a 2-m wave induces peak pressures consistent with literature values (e.g. Young et al. 2011; Thompson et al. 2019; Varley 2019), yet strains remain entirely elastic. This confirms that non-extreme wave impacts do not directly initiate damage, but can contribute to weakening through long-term fatigue (Brain et al. 2014). However, when parietal joints partition the cliff into columnar blocks, the dynamic response facilitates an overturning moment consistent with the toppling kinematics observed at Ventotene (Fig. 9c, d).

These results indicate that while individual high-energy wave impacts are not damaging on their own, repeated wave loading can contribute to long-term progressive destabilisation, and high-energy events may act as transient triggers once the rock mass has been sufficiently preconditioned (Lim et al. 2011). In this context, low-frequency/high-magnitude events, such as severe winter storms, can significantly accelerate the failure process (Earlie et al. 2015). These events often involve a synergy between exceptional hydrodynamic pressures and increased pore water pressure due to storm-related precipitation. This hydromechanical coupling can act as a definitive trigger, concentrated in a short timeframe, for rocky cliffs and structures (e.g. sea arches) already weakened by long-term fatigue. Such occurrences highlight how extreme meteo-marine conditions can dictate the precise timing of the final kinematic collapse, effectively condensing years of progressive degradation into a single high-energy event.

Under current Ventotene conditions, thermally induced strains are insufficient to initiate fracturing. Mechanical damage emerges only under intensified regimes or in more conductive lithologies (Fiorucci et al. 2018; Grechi et al. 2021), where the TAL deepens enough to overlap with pre-existing joints (Fig. 6; Fig. 7). In these scenarios, pre-existing cohesive joints experience downward-propagating failure (Fig. 11b), suggesting that thermal forcing can act as both a preparatory and a triggering mechanism in settings with pronounced seasonal variations (Alcaíno-Olivares et al. 2023; Gunzburger et al. 2005; Marmoni et al. 2020).

Realistically, simulation of progressive cliff degradation requires coupling basal undercutting with inward-migrating weathering, represented here through S–N fatigue-based strength degradation. This conceptual framework recognises that meteo-marine and climatic influences act in combination, rather than in isolation, to progressively weaken the cliff. Processes such as salt weathering, wetting-drying cycles, and thermally induced stress fluctuations (Furlani et al. 2014; Vann Jones (Née Norman) et al. 2015) operate at the cliff surface but progressively penetrate inward beyond the zones directly affected by waves or temperature variations. Implementing this progressive strength decay through S–N curves and coupling it with notch excavation enables the simulation to reproduce realistic sequences of coastal cliff failure (Fig. 10). Across all numerical configurations, weakening initiates at the cliff base and then migrates upward into the rock mass (Fig. 11b), reflecting the combined influence of basal erosion and surface-driven weathering in controlling the evolution of instability. The adoption of a fatigue-based S–N law represents a targeted step toward integrating the effects of sub-critical crack growth into our numerical framework. While the model operates at a macro-scale, this approach provides a physically consistent proxy for the progressive damage accumulation and stress corrosion processes—driven by seawater interaction and thermal cycling—that are known to govern rock mass weakening at stress intensities below peak resistance (Atkinson 1984; Nara et al. 2011; Grgic and Giraud 2014; Aldred et al. 2016). By embedding these micro-scale mechanisms into the strength-degradation law with FDEM approach, the proposed methodology bridges the gap between environmental multi-physics forcing and the global progressive failure of the cliff, offering a simplified but effective tool for simulating long-term rock mass degradation.

The simulations demonstrate that the progressive degradation of mechanical properties (*ϕ*, *c*, *σ*_t_) in the CCEs, together with the stiffness loss in the TFEs (Young’s modulus, *E*), leads to the isolation of rock blocks matching field observations at Punta Eolo (Fig. 11a). Notably, the FDEM framework reproduces counter-slope fractures naturally, without requiring predefined DFNs or unrealistic notch depths often invoked in previous studies (Styles et al. 2011; Calista et al. 2019; Su 2024).

The temporal calibration of the S–N approach was established by setting *N*_0_ to 100,000 mechanical steps, corresponding to the time required to excavate one NE (i.e. 25 cm), based on erosion rates estimated from Sunamura’s model for the Ventotene coast. This calibration represents approximately 5 years of basal undercutting under the documented wave climate and material properties. It implicitly assumes that surface weathering and basal erosion proceed at comparable long-term rates because both are driven by the same marine forcing. Importantly, the 100,000 cycles do not represent a literal count of discrete physical events, but rather a normalised measure of cumulative cyclic processes. Over a 5-year period, this metric integrates the effects of 1825 daily thermal cycles and thousands of energic wave impacts, each operating at different frequencies but contributing collectively to rock mass fatigue. Within this time span, simulations spanning approximately 200 years of cliff evolution (40 NEs) indicate that the system remains largely linear for the first roughly 80 years, after which nonlinearity emerges through the onset of basal block collapse. From that point onward, the recurrence of detachment events is primarily governed by the rate of mechanical property degradation, with the overturning of 2-m-wide rock blocks occurring at average intervals of approximately 40 years (Fig. 10e, f). While direct validation of these timescales remains limited by the short duration of the VFL monitoring, the predicted recurrence intervals fall within plausible ranges expected for Mediterranean tuffaceous cliffs and scaling can be interpreted as order-of-magnitude estimates rather than deterministic forecasts.

The analytical framework developed for Ventotene is readily transferable to other Mediterranean settings with comparable lithological and climatic conditions (e.g. Fazio et al. 2019; Mancini et al. 2017), where the proposed methodology could be directly extended.

Parametric TM analyses further suggest that thermal forcing becomes a dominant driver in more thermally responsive rocks (e.g. granites and basalts) or continental climates with high annual temperature amplitudes. However, the direct transposition of this framework to substantially different contexts has limitations. High-strength crystalline rock masses or coastlines exposed to extreme ocean swells would require site-specific recalibration of the S–N parameters and alternative temporal scaling. Applying the current calibration to environments with radically different erosion rates or loading frequencies could lead to a significant misrepresentation of both failure mechanisms and predicted timescales.

## Conclusions

This study demonstrates that the instability of soft rocky coastal cliffs, such as those of Ventotene, cannot be numerically explained by single controlling factors acting in isolation, but instead emerges from the coupled interaction between structural predisposition, marine undercutting, weathering-driven degradation, and time-dependent mechanical weakening. The FDEM framework adopted here proved capable of reproducing PRF phenomena only when basal erosion was explicitly coupled with rock strength degradation, highlighting the preparatory role of marine and climatic forcings in preconditioning the cliff system to collapse. Wave impacts and thermal forcing, when acting under present-day climatic conditions, were shown to induce predominantly elastic responses, while their contribution becomes mechanically relevant only in combination with long-term degradation processes, under more extreme thermal regimes or when considering different rock types.

Within this framework, the integration of CFD and FDEM represents an exploratory multi-modelling approach aimed at resolving the mechanical response of coastal cliffs to realistic environmental boundary conditions. Rather than reproducing wave-driven failure mechanisms directly, the coupled CFD-FDEM analyses provide quantitative insight into stress transmission, displacement patterns, and fracture-wave interactions under cyclic and sub-critical loading. These results indicate that sea-wave impacts primarily act as preparatory agents, contributing to progressive fatigue and sub-critical crack growth, and become mechanically relevant only as the system approaches marginal stability.

The cumulative influence of marine and thermal forcing was effectively captured through an S–N degradation approach, which offers a unified representation of damage accumulation induced by cyclic processes acting at different frequencies and magnitudes. By focusing on progressive strength decay rather than instantaneous loading, this framework enables heterogeneous environmental forcings to be consistently integrated and allows PRF mechanisms to emerge naturally within the FDEM simulations.

Beyond the Ventotene case study, the methodological framework presented here provides a transferable basis for investigating coastal cliff instability in other tuffaceous and thermally sensitive lithologies, and more generally for exploring how multiple environmental stressors interact across temporal scales to govern coastal rock slope evolution. Future developments should focus on refining temporal calibration through extended monitoring and on explicitly representing sub-critical crack growth induced by repeated wave loading, thereby strengthening the link between field observations and numerical modelling within a digital-twin perspective.

## Supplementary Information

Below is the link to the electronic supplementary material.

ESM1 (DOCX 784 KB)

## Data Availability

The data underlying this study are available upon request to the corresponding author.
