# Supplementary material for: Numerical modelling of static and dynamic factors controlling the progressive retreat of rocky coastal cliffs
Source: Landslides. 2026 Jun 27;23(10):2909–31. doi: 10.1007/s10346-026-02800-2 (PMC13574890; doi:10.1007/s10346-026-02800-2)
Supplement: Supplementary file 1 [file 10346_2026_2800_MOESM1_ESM.docx]

SUPPLEMENTARY MATERIAL FOR

**Numerical modelling of static and dynamic factors controlling the progressive retreat of rocky coastal cliffs**

Feliziani F.^1^, Marmoni G.M. ^1^*, Montagnese M . ^1^, Sobhani R.^2^, Istrati D.^2, 3,4^, Eberhardt E.^5^ & Martino S.^1^

^1^Sapienza University of Rome, Rome, Italy

^2^National Technical University of Athens, Athens, Greece

^3^Technical University of Braunschweig, Braunschweig, Germany

^4^Aarhus University, Aarhus, Denmark

^5^The University of British Columbia, Vancouver, Canada

*Corresponding Author. Email address: [gianmarco.marmoni@uniroma1.it](mailto:gianmarco.marmoni@uniroma1.it)

# **Introduction**

This document provides supplementary material regarding the theoretical and methodological framework of the research. Specifically, it offers a more detailed description of the Finite-Discrete Element Method (FDEM) and Computational Fluid Dynamics (CFD) approaches compared to the main paper. Furthermore, it details the methodological procedure derived from Sunamura (1992), which was used to define a notch width representative of the specific site conditions in the study area. Finally, the thermal inputs used in the thermomechanical analyses are also shown.

**Principles of the Finite–Discrete Element Method (FDEM)**

In the Irazu code framework (Geomechanica Inc., 2024), the modelling domain is discretised into triangular finite elements (TFEs) connected by cohesive crack elements (CCEs). The TFEs capture the elastic or elasto-plastic deformation of the intact rock continuum, whereas the CCEs are used to capture fracture initialisation and propagation within the fracture process zone (FPZ) of the continuum (Barenblatt, 1962; Dugdale, 1960) (Fig. 1a, b). For isotropic material behaviour, fracture initiation occurs when the opening displacement (*O*) or tangential shear slip (*S*) across a CCE exceeds the critical threshold (*O_p_* or *S_p_*) corresponding to the tensile (*f_t_*) or Mohr-Coulomb shear strength (*f_s_*) of the interface (Irazu, 2024). When the CCE opens beyond *O_p_* or slips beyond *S_p_*, the normal (*s*) or tangential (*t*) tractions gradually decrease until residual values (*O_r_* or *S_r_*) are reached, at which point a traction- or friction-free discontinuity is inserted, and a physical crack develops. Mode I tensile fracture initiation occurs when *f_t_* is exceeded (Fig. 1c), Mode II shear when the *f_s_* is exceeded (Fig. 1d), and a mixed-mode fracture initiation arises from their combined action (Fig. 1e). The evolution from peak to residual strength is governed by the fracture energies G_I_ and G_II_, which represent the energy per unit area required to propagate a crack in Mode I and Mode II, respectively (Hillerborg et al., 1976).

Contact stiffnesses for both TFEs and CCEs are controlled by penalty coefficients (Mahabadi et al., 2012; Munjiza and Andrews, 2000). In particular, normal (*p_n_*) and tangential (*p_t_*) contact penalty coefficients define the inter-element stiffness, while the fracture contact penalty coefficient (*p_f_*) defines the CCE stiffness.

To account for the energy dissipation due to non-linear material behaviour, a viscous damping factor (β) is assigned to the domain (Munjiza, 2004). For quasi-static problems, a β = 1 is recommended to dissipate unwanted dynamic oscillations, while, for dynamic problems (such as wave propagation) β is related to the seismic quality factor (Q) of the rock as β = 1/Q.

**Fig. 1** (a) Sketch of the fracture process zone (FPZ); (b) Numerical representation of the theoretical fracture propagation zone (FPZ) modelled in Irazu; Constitutive behaviour of the crack elements: (c) normal bonding stress versus crack opening for pure compressive/tensile loading (Mode I); (d) shear bonding stress versus crack slip for pure shear loading (Mode II); (e) criterion for mixed-Mode fracture initiation (mod. after Lisjak et al. 2013)

The fracture parameters were defined using empirical relationships derived from the literature. In particular, the normal (*p_n_*), tangential (*p_t_*) and fracture (*p_f_*) penalty coefficients were set to ten times the Young’s modulus, following guidelines provided in the Irazu Theory Manual (Irazu, 2024). Since no direct tensile test data was available, the fracture energies G_I_ and G_II_, were estimated according to Whittaker et al. (1992):

$$G_{I}= \frac{K_{IC}^{2}}{E'}$$

**Eq. 1**

where $E^{'}=E/\left( 1-v \right)^{2}$, with $E$ = Young’s modulus of the rock matrix, and $K_{IC}$ is the Mode I fracture toughness defined by Zang (2002) as follows:

$K_{IC}^{2}=f_{t}/6.88$

**Eq. 2**

where $f_{t}$ is the tensile strength.

Mode II fracture energy was taken to be ten times higher than the Mode I energy (Irazu, 2024).

# **The technical process to define a notch width representative of the coastal retreat rate characterising the cliffs at Punta Eolo**

The relationship proposed by Sunamura (1992) was used to define a notch width representative of the coastal retreat rate characterising the cliffs at Punta Eolo. In this formulation, the forces driving notch development are expressed as the ratio between assailing (Fw) and resisting (Fr) forces. The assailing forces include the hydraulic, abrasive and wave actions exerted by sea-wave impacts on the cliff, while the resisting forces are primarily controlled by the strength of the rock mass. According to Sunamura (1977), the controlling equation for notch development relates Fw and Fr as:

$$R=k\ln\left( \frac{Fw}{Fr} \right)$$

**Eq. 1**

$$Fw=E\rho gH$$

**Eq. 2**

$$Fr=ISc$$

**Eq. 3**

where $R$ is the notch retreat rate, which can be assumed equivalent to the cliff retreat rate, $k$ is a corrective constant with the same unit of $R$, $H$ is the wave height at the cliff toe, $\rho$ is the water density, $Sc$ is the compressive strength of the cliff material, and $E$ and $I$ are dimensionless coefficients representing the efficiency of abrasive beach sediment and weathering-induced weakening, respectively. Cliff retreat occurs when F_w_ exceeds F_r_. Substituting Eq. 4 and Eq. 5 into Eq. 3 yields:

$$R=k\left[ \Gamma+ \ln\left( \frac{\rho gH}{Sc} \right) \right]$$

**Eq. 4**

where $\Gamma$ is a factor relating the efficiency of abrasive beach sediment (E) and weathering-induced weakening (I):

$$\Gamma= \ln\left( \frac{E}{I} \right)$$

**Eq. 5**

$E$ is a function of the beach elevation relative to the mean sea level. Although Punto Eolo lacks a beach, hydraulic erosion remains active; therefore $E$ was assumed to be 5x10^4^ (Kline et al., 2014). An appropriate $\Gamma$ value for Punta Eolo can be determined from Eq. 8, assuming a UCS strength of 5 MPa (Fig. 2).

**Fig. 2** Plot of wave height at the cliff face (H_cliff_) versus average retreat rate (R) for Punta Eolo cliff at Ventotene. The contours represent the $\Gamma$ value required for the corresponding R value according with Eq. 4.

By considering a typical retreat rate range between 0.05-1.28 m/yr for analogous tuffaceous coasts, and an ordinary significant wave height of 0.2 m characteristic of Ventotene, a Γ value of 7.894 can be assumed representative for the Punta Eolo cliff. This value constrains the notch retreat rate at Punta Eolo to approximately 0.05 m/yr.

# **Principles and numerical model setup of the Computational Fluid Dynamics (CFD) modelling**

The OpenFOAM v2406 solver discretises the Navier-Stokes equations using the Finite Volume Method (FVM) (Eymard et al., 2000), resolving the transient and impulsive spikes in pressure on the cliff face with high fidelity. On this basis, the mass conservation is ensured through the continuity equation:

$$\nabla\cdot u = 0$$

**Eq. 8**

while the momentum equation, derived from Newton’s second law and expressing conservation of momentum, is given by:

$\frac{\partial u}{\partial t}+u\cdot\nabla u= -\frac{1}{\rho}\nabla p+\upsilon\nabla^{2}u+g$

**Eq. 9**

where $u$ is the velocity vector, $\rho$ is the water density, $p$ is the pressure, $\upsilon$ is the kinetic viscosity, and $g$ is the gravitational acceleration.

Since the problem involves two immiscible phases (air and water), the interFOAM solver was adopted, which applies the Volume of Fluid (VOF) method (Cifani et al., 2016; Hirt and Nichols, 1981) to track the free-surface interface. The transport equation of the phase fraction is governed by:

$$\frac{\partial\alpha}{\partial t}+\nabla\cdot(\alpha u )+\nabla\cdot(\alpha(1-\alpha)u_{r}) = 0$$

**Eq. 10**

where $\alpha$ is the volume fraction ($\alpha$ = 0 for air, $\alpha$ = 1 for water, and 0<<1 at the interface), and $u_{r}$ is an artificial compression velocity used to maintain a sharp interface between phases.

These equations, together, allow detailed resolution of wave impact processes and pressure along the cliff.

A Courant–Friedrichs–Lewy (CFL) number of 0.5 was imposed to guarantee accurate wave propagation and reliable interaction with the solid boundary, consistent with the recommendations of previous studies (Chen et al., 2014; Katsidoniotaki and Göteman, 2022). The computational mesh was divided into five regions, each characterised by a different resolution (Fig. 2b of the original paper). This zoning strategy ensured numerical accuracy while minimising computational cost. Mesh refinement was concentrated in areas where hydrodynamic interactions are most intense and sensitive to grid resolution. In particular, Region 1 was vertically stretched, with cell heights gradually decreasing from 10 cm at the seabed to 5 cm to match the cell size of Region 2 at its upper boundary. Regions 3 and 4 employed a constant cell height of 10 cm. For Region 5, located in front of the cliff, cells are refined by half in both directions to more accurately capture wave-structure interactions. A fixed cell length of 10 cm was applied to the entire domain length, providing an aspect ratio of 1 to 2, which has been proved accurate in wave-structure interaction problems (Ma et al., 2019; Wang et al., 2019).

Since the goal of this study is to evaluate the mechanical response of the cliff under energetic wave loading, the simulations adopted a representative sea wave height of 2 m, corresponding to rough-sea states affecting the study area, with a wave period of 4 s. This choice is justified by available observations at Punta Eolo, where fair-weather conditions typically involve significant wave heights of 0.2 m, but energetic events with heights up to 2 m have been recorded (Fig. 3a). In particular, during the monitoring period carried out by the VFL system, wave heights (Hmax) greater than 2 m were recorded 1240 times (Fig. 3b), demonstrating the statistical representativeness and recurrence of such energetic wave conditions within the local wave climate

**Fig. 3** (a) Bar plot showing the distribution of the maximum recorded sea wave heights (Hmax) measured by the VFL monitoring system at Punta Eolo over the entire observation period; (b) Zoomed bar plot focusing on energetic wave events, restricted to Hmax > 2 m

# **1D semi-infinite solid solution of Carslaw and Jaeger (1959) and Thermal inputs used in the thermomechanical analyses**

Thermal propagation into the cliff face was modelled using the 1D semi-infinite solid solution of Carslaw and Jaeger (1959), which allows for the quantitative evaluation of the temperature profile ($T$) in depth ($x$) in the case of a periodic thermal forcing:

$$T\left( x, t \right)=T_{0}+Ae^{-(x/\delta)}\sin(\omega t-x/\delta)$$

**Eq. 1**

with:

$$\delta= \sqrt{\frac{2a}{\omega}}$$

**Eq. 2**

and:

$$a= \frac{\lambda}{\rho C_{p}}$$

**Eq. 3**

where $\delta$ is the Typical Penetration Depth (TPD), that represents the distance to which a thermal front can propagate within the solid medium, $a$ is the thermal diffusivity of the medium, $\omega$ is the angular frequency of the temperature fluctuation, and $\lambda$, $C_{p}$ and $\rho$ are the thermal conductivity, specific heat capacity and relative density of the solid medium, respectively.

At depth $\delta$, the temperature amplitude decays to $1/e$ of the surface value. The TPD can therefore be interpreted as a theoretical parameter that quantifies the characteristic length scale of the exponential attenuation of thermal oscillations. The portion of the rock mass where temperature variations remain appreciable over time is commonly referred to as the Thermal Active Layer (TAL) (Gischig et al., 2011; Marmoni et al., 2020). Its thickness corresponds to the part of the medium effectively influenced by periodic thermal fluctuations and typically spans multiple TPDs, depending on the temperature threshold considered. In the present study, no specific threshold was imposed; thus, the TAL in this case defines the entire region experiencing measurable temperature variations.

**Fig.** **4** Time series of (a) daily, (b) annual, and (c) composite thermal periodic inputs. The composite signal was obtained by superimposing the daily temperature cycles onto the annual cycle. (d) Comparison between real and increased (+10 °C and +20 °C) composite thermal inputs used in the TM modelling

# **BIBLIOGRAPHY**

Barenblatt, G.I., 1962. The mathematical theory of equilibrium cracks in brittle fracture. Advances in applied mechanics 7, 55–129.

Chen, L., Zang, J., Hillis, A.J., Morgan, G.C., Plummer, A.R., 2014. Numerical investigation of wave–structure interaction using OpenFOAM. Ocean Engineering 88, 91–109.

Cifani, P., Michalek, W., Priems, G., Kuerten, J.G., van der Geld, C., Geurts, B.J., 2016. A comparison between the surface compression method and an interface reconstruction method for the VOF approach. Computers & Fluids 136, 421–435.

Dugdale, D.S., 1960. Yielding of steel sheets containing slits. Journal of the Mechanics and Physics of Solids 8, 100–104.

Eymard, R., Gallouët, T., Herbin, R., 2000. Finite volume methods. Handbook of numerical analysis 7, 713–1018.

Hillerborg, A., Modéer, M., Petersson, P.-E., 1976. Analysis of crack formation and crack growth in concrete by means of fracture mechanics and finite elements. Cement and concrete research 6, 773–781.

Hirt, C.W., Nichols, B.D., 1981. Volume of fluid (VOF) method for the dynamics of free boundaries. Journal of computational physics 39, 201–225.

Irazu 2D - Theory Manual, 2024. Irazu 2D Geomechanical Simulation Software. V. 6.1. Theory Manual.

Katsidoniotaki, E., Göteman, M., 2022. Numerical modeling of extreme wave interaction with point-absorber using OpenFOAM. Ocean Engineering 245, 110268.

Kline, S.W., Adams, P.N., Limber, P.W., 2014. The unsteady nature of sea cliff retreat due to mechanical abrasion, failure and comminution feedbacks. Geomorphology 219, 53–67. https://doi.org/10.1016/j.geomorph.2014.03.037

Lisjak, A., Liu, Q., Zhao, Q., Mahabadi, O., Grasselli, G., 2013. Numerical simulation of acoustic emission in brittle rocks by two-dimensional finite-discrete element analysis. Geophysical Journal International 195, 423–443.

Ma, Y., Yuan, C., Ai, C., Dong, G., 2019. Comparison between a non-hydrostatic model and OpenFOAM for 2D wave-structure interactions. Ocean Engineering 183, 419–425.

Mahabadi, O.K., Lisjak, A., Munjiza, A., Grasselli, G., 2012. Y-Geo: new combined finite-discrete element numerical code for geomechanical applications. International Journal of Geomechanics 12, 676–688.

Munjiza, A., Andrews, K., 2000. Penalty function method for combined finite–discrete element systems comprising large number of separate bodies. International Journal for numerical methods in engineering 49, 1377–1396.

Munjiza, A.A., 2004. The combined finite-discrete element method. John Wiley & Sons.

Sunamura, T., 1977. A Relationship between Wave-Induced Cliff Erosion and Erosive Force of Waves. The Journal of Geology 85, 613–618. https://doi.org/10.1086/628340

Sunamura, T., 1992. Geomorphology of rocky coasts. Wiley.

Wang, D., Dong, S., Sun, J., 2019. Numerical modeling of the interactions between waves and a Jarlan-type caisson breakwater using OpenFOAM. Ocean Engineering 188, 106230.

Whittaker, B.N., Singh, R.N., Sun, G., 1992. Rock fracture mechanics. Principles, design and applications.

Zhang, Z., 2002. An empirical relation between mode I fracture toughness and the tensile strength of rock. International journal of rock mechanics and mining sciences 39, 401–406.
